# Random survival forest for predicting the combined effects of multiple physiological risk factors on all-cause mortality

**DOI:** 10.1038/s41598-024-66261-0

**Published:** 2024-07-06

**Authors:** Bu Zhao, Vy Kim Nguyen, Ming Xu, Justin A. Colacino, Olivier Jolliet

**Affiliations:** 1 School for Environment and Sustainability, University of Michigan, Ann Arbor, MI USA; 2 Department of Environmental Health Sciences, School of Public Health, University of Michigan, Ann Arbor, MI USA; 3 Department of Biomedical Informatics, Harvard Medical School, Boston, MA USA; 4 School of Environment, Tsinghua University, Beijing, China; 5 Quantitative Sustainability Assessment, Department of Environmental and Resource Engineering, Technical University of Denmark, Kongens Lyngby, Denmark

**Keywords:** Random survival forests, Survival tree, All-cause mortality, Physiological factors, Risk visualization, Biomarkers, Diagnostic markers, Prognostic markers, Risk factors, Medical research, Epidemiology

## Abstract

Understanding the combined effects of risk factors on all-cause mortality is crucial for implementing effective risk stratification and designing targeted interventions, but such combined effects are understudied. We aim to use survival-tree based machine learning models as more flexible nonparametric techniques to examine the combined effects of multiple physiological risk factors on mortality. More specifically, we (1) study the combined effects between multiple physiological factors and all-cause mortality, (2) identify the five most influential factors and visualize their combined influence on all-cause mortality, and (3) compare the mortality cut-offs with the current clinical thresholds. Data from the 1999–2014 NHANES Survey were linked to National Death Index data with follow-up through 2015 for 17,790 adults. We observed that the five most influential factors affecting mortality are the tobacco smoking biomarker cotinine, glomerular filtration rate (GFR), plasma glucose, sex, and white blood cell count. Specifically, high mortality risk is associated with being male, active smoking, low GFR, elevated plasma glucose levels, and high white blood cell count. The identified mortality-based cutoffs for these factors are mostly consistent with relevant studies and current clinical thresholds. This approach enabled us to identify important cutoffs and provide enhanced risk prediction as an important basis to inform clinical practice and develop new strategies for precision medicine.

## Introduction

Understanding the relative importance of risk factors and their combined effects on all-cause mortality is key for risk stratification and helping design targeted interventions^1,2^. However, little is known about the combined effects of multiple physiological factors on mortality risk^3,4^. Survival analyses based on left truncated and right censored data are often conducted using linear models such as Cox proportional hazards (CPH) model and its extensions. In our previous study, we used CPH models to assess non-linear associations between all-cause mortality and each physiological indicator. We did this by discretizing the physiological indicator into nine quantiles and by using a weighted sum of cubic polynomials (spline)^5^. While these models offer valuable insights into the relationship between individual risk factors and mortality, their ability to measure the effects of multiple factors is limited to additive relationships^6,7^. This limitation may restrict the capture of complex combined effects of multiple risk factors. In contrast, models such as survival trees and random survival forests (RSF) are alternatives. They help to identify the most influential factors leading to increased mortality risk^8^. These models can also account for complex correlations, detect interactions and non-linear associations between multiple risk factors^9,10^, and maintain high predictive power^11^. Such capabilities are crucial for providing needed information to guide clinical prioritization and improve patient outcomes through early interventions and tailored treatments.

Furthermore, it is crucial to visualize the combined effects of risk factors to enable effective risk stratification, providing a direct understanding of the intricate patterns of diverse factors. While RSF models are powerful tools to detect complex combined effects^12^, visualizing these effects has been limited to pair-wise effects^8,9,13–16^, which may not fully represent the full suite of effects. Thus, there is a need to develop visualization tools for RSF to show the combined effects of multiple factors beyond just two.

To address these needs, this study aims to use survival-tree based machine learning (ML) models to (1) characterize the combined effects between multiple physiological factors and all-cause mortality, (2) identify the five most influential factors and visualize their combined influence on all-cause mortality, and (3) compare the mortality cut-offs with the current clinical thresholds.

## Results

Using the National Health and Nutrition Examination Survey (NHANES), we included 17,790 adults who were eligible for follow-up to ascertain their death status and had complete data on the physiological indicators and demographic factors. The participants had a median age of 46 (Mean: 47.38, SD: 19.28) years old (y/o), of which 9145 (51.4%) were women and 1932 (10.9%) were deceased. eTable 1 and 2 in SI show the population characteristics and the distributions of each physiological indicator for the entire and final dataset.

### Identification of key physiological factors

**Figure 1:**
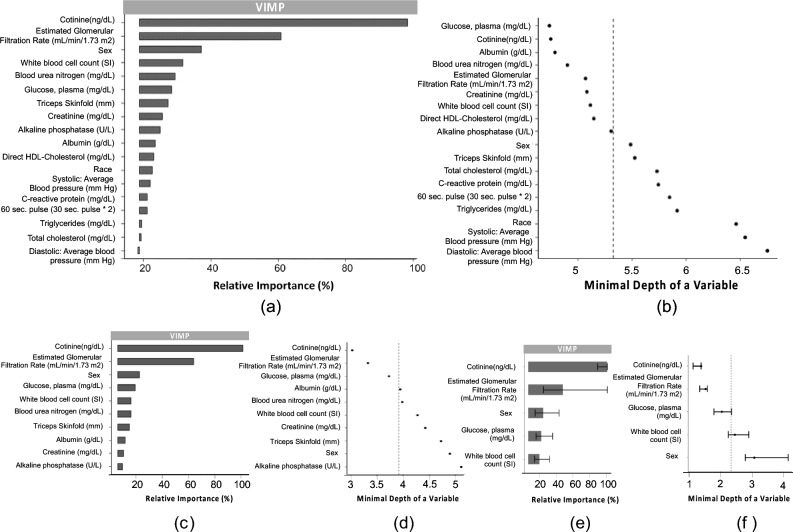
VIMP and minimal depth rankings for the RSF model using 18 (**a**, **b**), 10 (**c**, **d**), and 5 (**e**, **f**)) factors. The VIMP was scaled to 0–100% as a percentage of the top variable. The vertical dashed line in minimal depth plots was the mean of the minimal depth distribution used as the analytic threshold for evidence of variable impact. The concordance and sample size used in each model were (18 factors, 72.52%, 11,580), (10 factors, 71.09%, 12,045), and (5 factors, 70.12%, 17,790) respectively.

We used RSF models to select the key factors related to all-cause mortality based on variable importance (VIMP) and minimal depth rankings. As high collinearity may impede the identification of key variables due to the decrease in VIMP of correlated variables, we first constructed an RSF model using all 28 physiological variables and 2 demographical variables (9073 participants). We sorted the variables based on the VIMP (Supplementary information (SI), Fig. S1) and removed those highly correlated variables (Fig. S2) with relatively low VIMP to avoid the potential effects on the variable selection. Once the highly correlated variables were removed, we then conducted a recursive feature elimination procedure to identify the key physiological factors. The second RSF model used 18 factors (16 physiological factors and 2 demographic factors, 11,580 participants). The biomarker levels of cotinine and the GFR were the most dominant variables (Fig. 1a). Sex and white cell blood count had a VIMP around 20–25%, followed by blood urea nitrogen, plasma glucose, triceps skinfold, creatinine, and alkaline phosphatase, which had similar VIMPs between 10 and 20%. Figure 1b shows that the selection of variables according to the minimal depth was slightly different, while leaving the top 10 factors almost the same, except Direct HDL-Cholesterol which ranked 11th for VIMP. The VIMP was retained as the primary criterion for variable selection, as it is directly related to model performance. We repeated the selection procedure for the 10 most influential factors to build the third RSF model, with only slight changes in ranking, the top five factors according to VIMP remaining unchanged (Fig. 1c), but slightly different with the ranking from the minimal depth (Fig. 1d). We finally used these top five factors to construct the fourth RSF model for the final visualization, which includes cotinine, GFR, sex, plasma glucose, and white blood cell count (i.e., the key physiological factors). This dimension reduction procedure maintained the prediction power of the model as the concordance only slightly drops from 72.52 to 70.12% (Fig. 1e) while increasing the number of participants to 17,790. The bootstrap-based confidence interval (100 times) on the VIMP and the minimal depth metric of the top five factors showed that the minimal depth was more discriminant between variables than VIMP, while giving less importance to sex (Fig. 1f).

### Visualization of risk groups for key physiological factors using the survival tree

**Figure 2:**
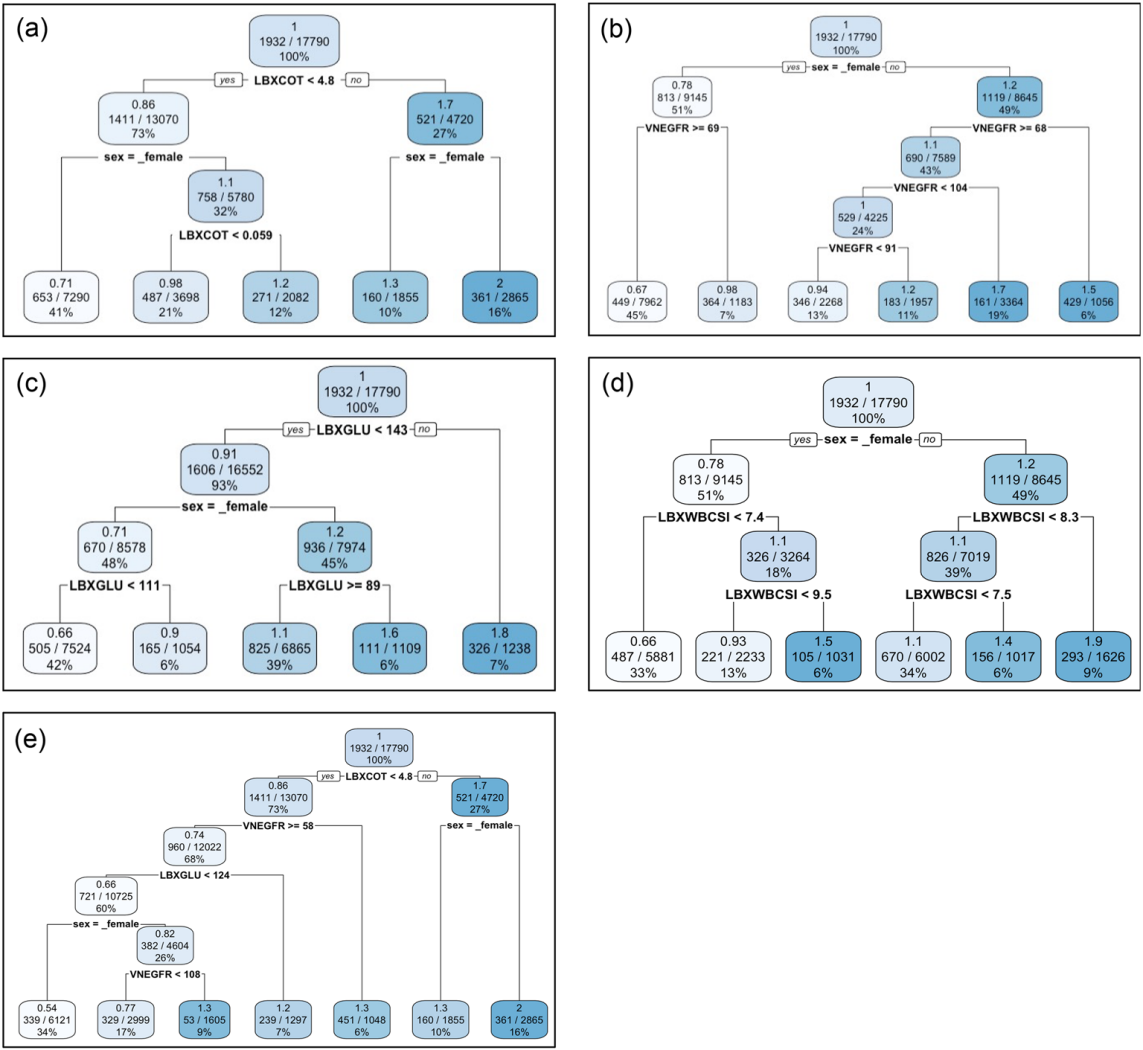
The identified risk groups for (**a**) serum cotinine, (**b**) GFR, (**c**) plasma glucose, (**d**) white blood cell count, and (**e**) all of these four factors together while adjusting for sex using survival tree models. Each node box presents three statistics, which from top to bottom are the relative risk, the events and sample sizes, and the proportion of this group to the total population.

In order to intuitively understand the stratification conducted by the survival-tree based models, we first used the survival tree model (i.e., a single tree) to present the identified risk groups for the key physiological factors (Fig. 2) selected based on the VIMP and minimal depth. The results for the other remaining factors are listed in Figs. S3–S26. We first evaluated the risk groups by each physiological factor solely while adjusting for different sex (Fig. 2a–d) to discover intricate effects of each factor. For all these identified key physiological factors, the separation between male and female is prominent as the first or second split in the tree structure, demonstrating their underlying difference in relative risks (0.78 against 1.2). For serum cotinine (LBXCOT, ng/mL), the survival tree (Fig. 2a) identified the threshold of 4.8 ng/mL between the high-risk group (smokers) and the low-risk group (non-smokers/passive smokers). For the low-risk male group, the tree was further split at 0.059 ng/mL, as the cut-off between non-smokers and passive smokers. For the GFR (VNEGFR, mL/min/1.73 m^2^) (Fig. 2b), low GFR (< 69 mL/min/1.73 m^2^) substantially increased the risk (0.98 against 0.67 for females, 1.5 against 1.1 for males). A similar situation also stood for males with a high GFR (> 104 mL/min/1.73 m^2^). The survival tree (Fig. 2c) of plasma glucose (LBXGLU, mg/dL) shows a substantially increased risk for high plasma glucose (> 143 mg/dL, 1.8 against 0.91). For the lower-risk group (plasma glucose lower than 143 mg/dL), the relative risk for females was further reduced for participants with plasma glucose lower than 111 mg/dL (0.66 versus 0.90). In contrast, the risk was increased for males who had plasma glucose lower than 89 mg/dL (1.60 versus 1.1). Risks were lower for females with low plasma glucose, but there was no significant sex difference in the high plasma glucose group. For white blood cell count (LBXWBCSI, Fig. 2d), there was an increased risk of high white blood cell count (> 7.4, 1.1 against 0.66 for females, 1.4 against 1.1 for males). Risk further increased at white blood cell count higher than 9.5 for females (1.5) and higher than 8.3 for males (1.9). When considering all of these key physiological factors together, only part of the dominant effects is observed in the tree structure (Fig. 2e). The survival tree still conducted the first split between the smokers and the non-smokers/passive smokers. For the non-smokers/passive smokers, low GFR (< 58 mL/min/1.73 m^2^) increased the risk for the subpopulation (1.3 against 0.74). For the low-risk group, the risk was further reduced for participants with plasma glucose lower than 124 mg/dL (0.66 versus 1.2). It is only then that sex showed some effect, but the difference between male and female is still significant (0.82 versus 0.54). Such results from a single tree are not fully stable as the tree structure changes with sample sizes (Fig. S27). However, most of the conclusions are still consistent across sample sizes, confirming the usefulness of the survival tree model for interpretation.

### Accurate quantification of mortality risk for multiple physiological factors using RSF

**Figure 3:**
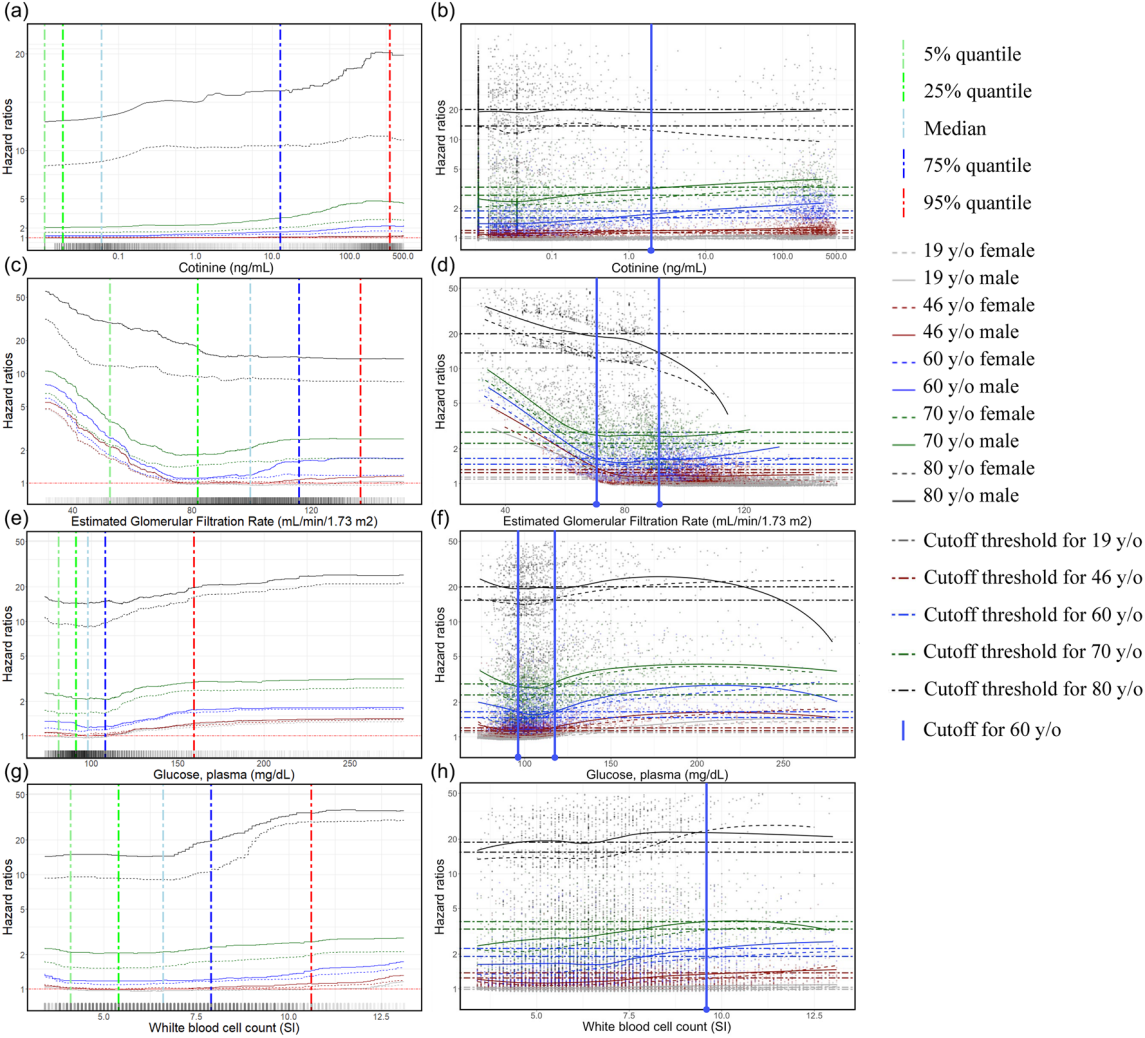
Predicted variation in overall HR for different age and sex groups as a function of each of the key physiological factors (**a**) cotinine, (**c**) GFR, (**e**) plasma glucose, and (**g**) white blood cell count using RSF, fixing the values of other factors at their median value. The vertical two-dashed lines represent the 5%, 25%, 50%, 75%, and 95% quantiles for each factor among the entire population. Predicted HR for each individual and the trend lines for each age- and sex- group in the NHANES subpopulation plotted based on each of the key physiological factors (**b**) cotinine, (**d**) GFR, (**f**) plasma glucose, and (h) white blood cell count without any constraints for other factors. Each point represents the HR of an individual compared to the reference person. The horizontal two-dashed lines represent the cutoff thresholds (10% increase of HR from the median person for each sex- and age- group) for each factor. The blue solid vertical line represents the identified mortality-based cutoffs.

Although a single survival tree is superior for visualization, the results from a single tree can be highly unstable (easy to overfit) and have less prediction power compared with RSF. Therefore, we calculated the hazard ratio (HR) and the HR_95%→5%_ (for example, the HR_95%→5%_ of cotinine for 80 s is calculated using 20.12 (the intersection between the red dashed vertical line with the fitted line of 80 s)/12.99 (the intersection between the green dashed vertical line with the fitted line of 80 s) = 1.55) for multiple physiological factors using RSF to achieve a more robust estimation. The left side of Fig. 3 presents the estimated HR as a function of each of the key physiological factors (i.e., fitted lines) and the values in Table 1 are the HR_95%→5%_ for different age and sex groups, which is controlled assuming a median value for the other key factors for different age and sex groups. As expected, HR increased with age, especially in the elder groups (> 60 y/o). Females always had a lower HR compared with males. And the increasing cotinine, plasma glucose, white blood cell count, and the decrease in GFR levels may increase the mortality risk (Table 1). Moreover, there were additional interesting age- and sex-specific results. For cotinine (Fig. 3a), as expected, the HR increased with the cotinine level and there were significant differences in HR between non-smokers (~ 0.02 ng/mL), passive smokers (~ 1 ng/mL), and smokers (> 300 ng/mL). Also, due to the survival bias, cotinine has more important effects in the 60 s and 70 s than in the 80 s (Table 1). For GFR (Fig. 3c), HR increased at a low GFR (< 70 mL/min/1.73 m^2^), especially for the 80 s (Table 1), which was consistent with the survival tree results. In this low GFR range (< 70 mL/min/1.73 m^2^), the slope of HR increase was also steep for younger people. In addition, HR also increased at GFR higher than 110 mL/min/1.73 m^2^, in particular for the groups of 60 and 70 y/o males, but not for 80 y/o. For plasma glucose (Fig. 3e), HR increased when plasma glucose increased above 110 mg/dL and reached its maximum around 150 mg/dL. The overall effects of plasma glucose are generally stable for males but increasingly important for females with age increasing. For white blood cell count (Fig. 3g), there was a strong effect in the elderly population when the white blood cell count was higher than 8 and the HR reached the maximum for a white blood cell count of around 10.5. These significant effects among the elderly population, especially in the 80 s, are also confirmed by the results provided in Table 1.

**Table 1:** HR_95%→5%_ ratio and their bootstrapping confidence interval for different age and sex groups.

Male				
Age group	Cotinine	Glomerular filtration rate	Glucose plasma	White blood cell count
80s	1.55 (1.45–1.63)	0.47 (0.45–0.55)	1.32 (1.19–1.44)	2.30 (1.92–2.44)
70s	2.34 (2.16–2.42)	0.70 (0.63–0.74)	1.32 (1.26–1.35)	1.23 (1.11–1.29)
60s	1.90 (1.70–2.00)	0.65 (0.57–0.70)	1.27 (1.20–1.34)	1.22 (1.14–1.30)
46s	1.18 (1.09–1.22)	0.53 (0.49–0.59)	1.22 (1.16–1.29)	1.09 (1.03–1.11)
19s	1.07 (1.04–1.09)	0.49 (0.48–0.51)	1.29 (1.18–1.42)	1.02 (1.01–1.11)

Female				
Age group	Cotinine	Glomerular filtration rate	Glucose plasma	White blood cell count
80s	1.35 (1.24–1.41)	0.73 (0.65–0.80)	1.72 (1.54–1.90)	3.02 (2.09–3.33)
70s	1.86 (1.78–2.00)	0.76 (0.70–0.80)	1.58 (1.49–1.65)	1.27 (1.19–1.37)
60s	1.54 (1.49–1.66)	0.56 (0.53–0.60)	1.39 (1.35–1.46)	1.15 (1.09–1.20)
46s	1.11 (1.07–1.15)	0.60 (0.50–0.69)	1.16 (1.10–1.29)	1.03 (1.01–1.06)
19s	1.04 (1.02–1.06)	0.57 (0.50–0.68)	1.21 (1.10–1.36)	1.02 (1.01–1.10)

Overall, the results in Fig. 3 were mostly in accordance with the individual results of the survival tree models, with slight differences attributable to the different treatments for the age and, thereby, the difference in concordance of various models. The statistical analysis demonstrated the superiority of the RSF models, with a concordance of 85.5%, over the survival tree models (one physiological factor for each time) with a concordance of 61.1% (cotinine), 62.6% (GFR), 61.6% (plasma glucose), and 64.8% (white blood cell count). When using all five factors in the model construction, the predicted variation in HR was also slightly different from the prediction made by the model using one single factor at a time (Fig. S28). These results demonstrated the need for studying the combined effects of multiple factors rather than single factors.

On the right side of Fig. 3, we also plotted the predicted HR for each individual and the trend lines for each age- and sex- group in the NHANES subpopulation based on each of the key physiological factors. This figure demonstrates the mortality risk change due to the change of a specific factor in the studied population without the constraints of other variables. Overall, the trend lines are mostly consistent with the fitted lines, although variations in other factors for each individual have introduced additional effects on HR, especially for elder groups with fewer observations. Based on this result, we could identify some important age-specific mortality-based cutoffs. Using 60 y/o groups as an example, for serum cotinine (ng/mL, Fig. 3b), we could identify a threshold around 2 ng/mL. Regarding GFR (mL/min/1.73 m^2^, Fig. 3d), the models suggested two cutoffs around 70 and 90. For plasma glucose (mg/dL, Fig. 3f), the model also identified two cutoffs, one around 96 and another one around 116. In terms of white blood cell count (Fig. 3h), our models detect an upper bound around 9.5. While, these cutoffs could vary significantly among different age groups.

### Visualization of the combined effects of key factors on mortality risks using RSF

**Figure 4:**
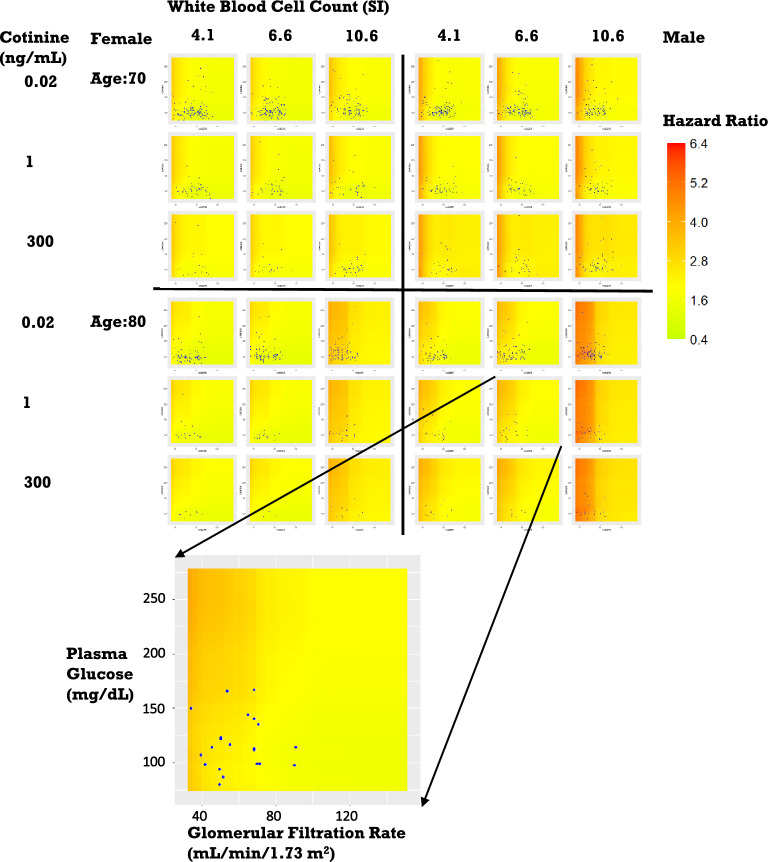
Combined effects of key factors for age groups of 70 and 80. Each 3 × 3 heatmap presented the continuous variation of HR from low (green) to high (dark red) as a function of GFR (X-axis) and plasma glucose (Y-axis) using RSF. These functions were then stratified by age and sex, and three different levels of white blood cell count and serum cotinine. The points in each heatmap represented the subpopulation for this specific stratification.

To comprehensively visualize the combined effects of the five most influential factors on mortality risks, Fig. 4 shows a series of heatmaps presenting the mortality risk across the range of different measurement values for GFR and plasma glucose using the RSF with stratification of two age groups, two sexes, three levels of white blood cell count, and three levels of cotinine levels. The heatmaps themselves highlight the combined effects of GFR and plasma glucose. For the elder population, there was a much stronger effect of GFR as there is a wider high-risk area for age groups of 80 compared with 70. Meanwhile, high plasma glucose had a more substantial effect at low than at high GFR. For younger ages, effects were mainly dominated by GFR alone (Figs. S29 and S30). White blood cell count had a much more substantial effect at 80 y/o than for younger ages. Besides, on the low and median levels of white blood cell count (4.1 and 6.6), there are strong combined effects for a high level of glucose plasma and a low level of GFR, while the combined effects decrease when the glucose plasma goes down. However, for a high level of white blood cell count (10.6), the combined effects are dominated by the GFR with no significant differences with the change of glucose plasma. For cotinine, smoking was associated with higher HR for the 70 y/o, but with moderate effect on the 80 y/o. For the younger group, there were similar effects as the 70 y/o.

## Discussion

Enhanced risk prediction can improve public health by identifying and prioritizing individuals at increased risk of an adverse outcome to relevant interventions. Here, we used two different models to quantify and visualize the effects of different factors on all-cause mortality: the survival tree model and the RSF model. The results demonstrated that these survival-tree based methods using multiple inputs can provide reasonable predictions of all-cause mortality risk in study participants recruited as part of NHANES, with a concordance index of up to 85.5%. We leveraged physiological factors in a diverse population with follow-up mortality data through linkage with the National Death Index. For individual physiological factors along with demographic information, the survival tree (i.e., a single tree rather than RSF with many trees) allowed us to split the population into different risk groups with similar survival behaviors. Once the variables and the hyperparameter of the survival tree are determined, the structure of the tree will be fixed and provide crucial clinical threshold information that is automatically detected by the model. Through the VIMP and minimal depth ranking, we identified the relative importance of physiological factors in predicting mortality, showing that cotinine level, GFR, plasma glucose, sex, and white blood cell count were the most influential ones. We further visualized the continuous relationships of physiological factors and their combined effects on mortality across individuals of different ages and sexes. Using our visualization tool, we identified age-specific mortality-based cutoffs using RSF, further confirming that these associations were rarely linear. This approach enabled us to predict HR for a given individual and identify important physiological factors as an important step and basis toward precision health of public health, providing needed information to guide clinical test prioritization and improve patient outcomes through early interventions and tailored treatments.

The predicted HRs provided by the RSF offer insightful risk cutoffs which are consistent with relevant studies and current clinical thresholds. For serum cotinine (ng/mL), the identified thresholds to distinguish the different types of smokers were mostly consistent with the results from Benowitz et al., (3.08)^17^ and Kim’s review (3–20)^18^. Regarding GFR (mL/min/1.73 m^2^), the lower cutoff (70) is slightly higher than the clinical practice guideline (60) and our previous study (64.6)^5^, while the upper bound is consistent. For plasma glucose (mg/dL), the identified lower cutoff further confirms our previous results (95.4)^5^ while the upper bound is slightly different (107.9). In terms of white blood cell count, our models only detect an upper bound around 9.5 which is aligned with the normal range^19,20^, while leaving the lower bound undetected. Specifically, we also found the distribution for each physiological factor and the identified thresholds could slightly vary among different age- and sex- groups. This was especially the case with GFR, where no upper threshold is found for the age group of 80. These results suggest, although general clinical thresholds are helpful, some age- and sex- specific thresholds are needed towards precision medicine.

There are a number of strengths in using the RSF approach to understand the relationship between multiple physiological factors and mortality risk. First, the RSF approach allows for the quantification of mortality risk from single and multiple physiological indicators. Second, due to the inherent flexibility in the RSF approach, we were able to quantify combined effects within and between physiological measures and mortality risk with higher prediction performance compared with traditional CPH models used in our previous studies^5^. Third, the RSF model provided clear rankings of which physiological indicators are most important in predicting elevated mortality risk. This importance of physiological factors can be used to help inform clinical practice (e.g., triaging) and interventions and develop new strategies for precision medicine. Fourth, we developed a heatmap-based method, which was the first to visualize the combined effects of multiple physiological factors and mortality risk across individuals of varying demographic characteristics. Although there are only moderate interactions witnessed in our case, this method provides the potential to quantify and visualize the high dimensional effects of multiple factors, which could facilitate the interpretation of the RSF models and streamline the risk analysis of individual participants based on their physiological factor measurements.

However, this study also had some limitations that should be addressed in future research. First, we used the provided models to predict the risk of all-cause mortality, rather than disease-specific mortality. We chose all-cause mortality over cause-specific mortality to ensure sufficient events to train the models. However, mortality due to different diseases is likely driven by the dysregulation of different biological systems, which would be reflected in different patterns of altered clinical biomarkers. Second, while we were able to take advantage of death certificate linkage to provide a longitudinal aspect to the otherwise cross-sectional NHANES study, future research should improve this approach by including serial measurements of the physiological indicators over time and tracking the changes in these measurements over time^21^. Third, we did not evaluate the impact of comorbidities on the mortality predictions and the absolute magnitude of the effect might be underestimated due to the underlying variability in factors that cannot be reflected by one point in time measurements. Future research should validate and expand on this approach specifically in well-characterized populations to ensure that the findings are applicable in a clinical setting. Fourth, although we have used correlation analysis and recursive feature elimination procedures to minimize the effects of collinearity that existed in the variables, the remaining collinearity could still slightly affect the interpretation of the results. In future studies, a domain- and disease- specific variable selection could be conducted to find specific interactions and combined effects for multiple predetermined interested factors. It is also important to find new metrics or methods to provide more robust and reasonable variable selections. Lastly, the interpretation of the ML-based model is still subjective and heavily depends on domain knowledge. Although the results are validated and could be explained by previous studies^10,22^, the lack of causality may lead to distrust of the provided results. In the future, we aim to build knowledge-based and theory-based ML models to further improve the characterization and interpretation of the results.

## Methods

### Study population

NHANES is a cross-sectional study to characterize the health of the non-institutionalized, civilian US population. For this study, we used the continuous 1999–2014 NHANES data on physiological factor measurements and demographics and merged these data with the National Death Index (age >  = 18) to ascertain mortality information collected through December 31, 2015^23^. We excluded participants who did not have data on mortality status (N = 37,046) and those who were not followed up (N = 13), yielding a final sample of 45,032 with 28 physiological factors and mortality information. During the model training, different subsets of the sample are used based on the different variable selection and the completeness of the measurements. Supplementary text 1 (SI1) and Fig. S31 in the SI provide more information about the curation procedures.

### Survival tree

Survival tree is a popular nonparametric ML model for classification and regression of censored survival data^24^. The model has a hierarchically organized structure (i.e., tree-liked structure), constructed through rounds of recursive binary partitioning (i.e., split the feature space into binary parts based on the selected variable). Through the stratification conducted from the tree structure, the population is split into different risk groups with similar survival behaviors and the potential clinical threshold can be automatically detected by the survival tree model. Here, we used survival trees to visualize the risk groups of each physiological factor and all physiological factors together in the NHANES population. Once the variables and the hyperparameter of the survival tree are determined, the structure of the tree will be fixed. We used mortality status as the event outcome and age as the time scale to avoid the overwhelming effect of age. More details of the model and the hyperparameter setting can be found in SI2.

### Random survival forests

Although a single survival tree has superiority for visualization, its results can be highly unstable and sensitive to overfitting. The RSF draws several bootstrap samples from the original dataset to build many new survival trees (i.e., a forest). By averaging the results over all trees, the model is more robust to outliers and provides a more reliable measure to predict time to event. At each candidate split in the tree structure, a random subset of features was tested to create the split to decorrelate the results among these trees. In this study, we used RSF models to quantify the combined non-linear and age-specific associations between all-cause mortality and multiple physiological factors. In this study, we calculated the HR and the HR_95%→5%_ (the ratio between the target variable changing from its 95% quantile to 5% quantile). The time to event was used as the time scale and the male with the median level of the selected physiological factor and age was retained as the reference group with a HR of 1.0. More details of the model and the hyperparameter setting can be found in SI3.

### Identification and selection of key factors

There are typically two metrics to demonstrate the variable importance, which are the relative VIMP and the minimal depth. A high VIMP indicates a higher predictive power of the variable and a low minimal depth represents the variable will frequently appear at the top of the root node to partition large samples of the population. In order to identify the influential factors that may be masked by the overwhelming effect of age (Figs. S32 and S33), the models constructed for variable selection used age as the time scale rather than the time to event^25–27^. As the collinearity may impede the identification of key variables due to the decrease in VIMP of correlated variables^28–31^, we removed those highly correlated (absolute Pearson correlation coefficient > 0.6) physiological factors with relatively low VIMP in the full model (Fig. S1). In order to minimize the remaining collinearity and identify those truly important factors, we then conducted a recursive feature elimination procedure, which performed feature selection by iteratively training a model, ranking features, and then removing the lowest-ranking features^30,31^. After three rounds of elimination, we removed those factors with relatively low VIMP and five key factors were identified and applied in the final model.

### Visualization of the combined effects of the top five influential factors

To analyze the combined effects of the top five factors of interest on all-cause mortality, we applied the previously constructed RSF model to a grid of values covering all possible combinations of these factors. Their combined effects on mortality were visualized by presenting 3-by-3 bidimensional heatmaps of HR for each age and sex as a function of the two influential factors (e.g., glomerular filtration rate (GFR) and plasma glucose level), for three values of two additional factors (e.g., low, medium, and high levels of white blood cell counts (5% (4.1), 50% (6.6), and 95% (10.6) quantiles) and cotinine level (0.02 (non-smoker), 1 (passive smoker), and 300 (smoker) based on the guidelines of cotinine values for smoking status classification^17,18^).

### Ethics approval and informed consent

The National Center for Health Statistics research ethics review board provided ethical approval of the study. The study was conducted in accordance with the relevant guidelines and regulations. All participants provided written informed consent.

### Supplementary Information

Supplementary Information.

## Data Availability

Data for the study was collected by the Centers of Disease Control and Prevention and our curated data is publicly available on Kaggle (https://www.kaggle.com/datasets/nguyenvy/nhanes-19882018?select=dictionary_nhanes.csv), figshare (https://figshare.com/articles/dataset/NHANES_1988-2018/21743372), and Hugging Face (https://huggingface.co/datasets/nguyenvy/cleaned_nhanes_1988_2018). The analytic code used in this report is publicly available on GitHub (https://github.com/zhaobuterry/Random-Survival-Forest-for-Predicting-the-Combined-Effects-of-Multiple-Physiological-Risk-Factors).
