# Supplementary material for: Random survival forest for predicting the combined effects of multiple physiological risk factors on all-cause mortality
Source: Sci Rep. 2024 Jul 6;14:15566. doi: 10.1038/s41598-024-66261-0 (PMC11227534; doi:10.1038/s41598-024-66261-0)
Supplement: Supplementary file 1 — Supplementary Information. [file 41598_2024_66261_MOESM1_ESM.docx]

**Supporting Information for**

Random Survival Forest for Predicting the Combined Effects of Multiple Physiological Risk Factors on All-Cause Mortality

Bu Zhao^1,^*, Vy Kim Nguyen^2,3^, Ming Xu^4^, Justin Colacino^2^, Olivier Jolliet^2,5,^*

^1^ School for Environment and Sustainability, Univ. of Michigan, Ann Arbor, MI, USA

^2^ Dep. of Environmental Health Sciences, School of Public Health, Univ. of Michigan, Ann Arbor, MI, USA

^3^ Dep. of Biomedical Informatics, Harvard Medical School, Boston, MA, USA

^4^ School of Environment, Tsinghua University, Beijing, China

^5^ Quantitative Sustainability Assessment, Department of Environmental and Resource Engineering, Technical University of Denmark, Kgs. Lyngby, Denmark

* Olivier Jolliet and * Bu Zhao

**Email:**  [ojolliet@umich.edu](mailto:ojolliet@umich.edu) or [zhaobu@umich.edu](mailto:zhaobu@umich.edu)

**This PDF file includes:**

Supporting text

Figures S1 to S33

Tables S1 to S2

R code used for data analysis

SI References

Supporting Information Text

**SI1.** **Curation procedures.** We identified 60 biomarkers and measures that characterise physiological function. In NHANES, not all physiological indicators are measured in all study participants. As such, many participants with mortality data do not have measurements for some physiological indicators. Thus, we excluded physiological indicators that have low overlap with mortality data by excluding those with measurements in fewer than six NHANES cycles (n=10) and with a sample size of less than 10 000 participants (n=21). As we focused on continuous variables for studying linear and non-linear associations, we also excluded physiological indicators that are categorical (n=2). The final dataset for analysis consisted of 27 physiological indicators. Laboratory methods used to measure the physiological indicators are provided on the NHANES Laboratory Data website.

**SI2.** **Survival tree.** Survival tree is a popular nonparametric model for analyzing survival data introduced by Gordon and Olshen in 1985^[1]^. It is an extension of the classification and regression tree (CART) model^[2]^ and is designed for classification and regression purposes of censored survival data. The model has a hierarchically organized structure (i.e., tree-liked structure), constructed through rounds of recursive binary partitioning (i.e., split the feature space into binary parts based on the selected variable). Through the stratification conducted from the tree structure, the model can automatically detect interactions between individual risk factors. Each resulting subspace is called as a “node” and those nodes in the final partition are called “terminal nodes” or “leaf nodes”. For each split, the log-rank test (see details in SI4) has been conducted on all the combinations of possible variable ***x*** and split point ***c*** in order to have participants with similar survival behavior based on their covariates. The larger the absolute value of the log-rank statistic, the greater the difference between the survival curves between each subgroup and the greater the node separation. The retained split is the one with the largest log-rank test value which guarantees the best separation of the sub-groups based on the median survival times^[1, 3]^. By repeating such procedures, the sample space is narrowed into smaller sub-groups, containing participants with similar relative risks^[1, 4]^. In this study, the stopping rule of the tree was that at least 1000 observations (around 5% of the population) must be included in a terminal node and any split that did not decrease the overall lack of fit by a factor of 0.00001 (i.e., the complexity parameter) will not be attempted. In the figure of each survival tree, the node presents three statistics, which from top to bottom, are the relative risk for this group, the events/sample size of the group, and the proportion of this group to the total population. At each split, the left branch is the group that meets the criterion shown under the node. In this study, we set non-Hispanic white male with the median level of the selected physiological factor and age as the reference group, where the selection of median value is mainly due to the skewed distribution of the factors. The above analysis was conducted using the R packages “LTRCtrees” and “rpart”.

**SI3.Random survival forests (RSF).** Although a single survival tree is superior for visualization and has potential for modeling non-linear interactions, results from a single tree can be highly unstable (easy to overfit) and have less prediction power compared with RSF. The RSF draws bootstrap samples from the original NHANES dataset (i.e., randomly select participants with replacement of the same sample size) and then uses this dataset to build a new survival tree model. At each candidate split in the tree structure, a random subset of features (i.e. only select part of the factors as predictors, mtry = 3 in our study) was tested to create the split. This procedure was repeated ***n*** times in order to grow a single tree into a forest (e.g., tree size of 500). In this process, at least 200 observations are required for each node in the tree. By averaging over all trees, the model is more robust to outliers and a more reliable measure (with low bias and moderate variance) to predict time to event^[5, 6]^. The direct output of the RSF model includes cumulative hazard function (CHF) - which represents the total risk cumulated up to time ***t***, the predicted survival - which represents the probability of surviving to time ***t***, and the predicted ensemble mortality - which can be interpreted as the number of deaths that would be expected given that all other subjects had the same predictor values. Since bootstrapping involves random selection of participants to build a training set, the remaining non-selected part (i.e., out-of-bag (OOB) data, usually account for 1/3 of the data) could then be used as the test set. The model performance was evaluated by the OOB prediction error rate calculated as one minus concordance, where concordance (also known as C-index) is defined as the proportions of pairs for which the order of survival times is matched by the order of model predictions. To identify key physiological factors related to all-cause mortality, we constructed the RSF model using all remaining 16 physiological factors after removing the highly correlated factors and 2 demographic factors (i.e., race and sex).

In this study, to characterize how mortality risk changes with specific physiological factors, we further calculated the hazard ratio (HR) using the predicted ensemble mortality (prognostic index in the context of the CPH regression model) which normalized between the lowest (at 0) and the highest scoring individual (at 1)^[7]^. The HR was then calculated by performing a CPH regression with the only predictor as the normalized ensemble mortality. HR was then equal to $\boldsymbol{e}^{\beta\boldsymbol{\cdot}\boldsymbol{x}_{\boldsymbol{norm}}}$ with $\boldsymbol{\beta}$as the coefficient of the CPH regression and $\boldsymbol{x}_{\boldsymbol{norm}}$ the normalized ensemble mortality. We then visualized the non-linear relationship between HR and each physiological factor for fixed values of the other physiological or demographic factors. The above analysis was conducted using the R packages “randomForestSRC”, “ggRandomForests”, and “survival”.

**SI4.** **The relative variable importance (VIMP) and the minimal depth.** The VIMP ranks the most important variables according to their impact on the predictive ability of the forest. The VIMP for a specific variable in the model is the difference in OOB error between the original observed data and the randomly permuted data^[8-11]^. A low VIMP indicates the low predictive power of that variable. The minimal depth was obtained by inspection of the forest construction to rank variables based on the assumption that the variables with high impacts on the prediction are those that most frequently split nodes nearest to the trunks of the trees (i.e. at the root node). A low minimal depth represents the variable partitions large samples of the population. After three rounds of elimination, five key factors were identified and the model applied to the entire dataset of 17,790 participants (from all eight survey cycles except cycle 2 which lacking the measurements for the glomerular filtration rate (GFR)) who had measurements for all of these five key factors.

**SI5.Log-rank test on survival tree model.** For a split on a continuous predictor defined as ***x ≤c*** and ***x > c,*** let ***t_1_ < t_2_ < … < t_m_*** be the distinct event times in the parent node ***h.*** Let ***d_k,l_*** and ***Y_k,l_*** be the number of events and subjects at risk at time ***k*** in the left child node, respectively, and let ***d_k,r_*** and ***Y_k,r_*** be the same for the right child node. Let ***Y_k_*** and ***d_k_*** be the total number of subjects at risk and the total number of events at time ***k***, respectively. Then, the log-rank statistic for the split point on the variable ***x*** is given by:

$$L\left(c,x\right)=\frac{\sum_{k=1}^{m} \left(d_{k,l}-Y_{k.l}\frac{d_{k}}{Y_{k}}\right)}{\sqrt{\sum_{k=1}^{m} \frac{Y_{k,l}}{Y_{k}}\left(1-\frac{Y_{k,l}}{Y_{k}}\right)}\left(\frac{Y_{k}-d_{k}}{Y_{k}-1}\right)d_{k}}$$

The larger the value of ***|L(c, x)|***, the greater the difference between the survival curves and the greater the node separation. The objective is to find a best variable ***x*** with an optimal split point ***c***.

**SI6.Confidence Interval.** To consider the effects of uncertainty from the model and training data, we further calculated the confidence intervals using the bootstrap method (resampling the training set 100 times)^[21]^ at certain key points. As shown in Fig. 4(b) in the main manuscript, we selected for 70 y/o male a low GFR (i.e., 5% quantile, the green circle), a median GFR (i.e., the overall median, the sky blue triangle), and a high GFR (i.e., 95% quantile, the red star). The point estimate and the 90% confidence interval for the ratio between the lower value and the median HR value is centered at 1.74 (1.02, 2.24) and for the ratio between higher and median HR value at 1.23 (1.12, 1.25). As expected, the confidence interval for extreme values of GFR was high but still significant, with ratios significantly higher than 1, which demonstrates the robustness of the prediction results.

**SI7.** **Personal Hazard Ratio Estimator.** The RSF approach can be further personalized, and the ***Personal Hazard Ratio Estimator*** provided in <https://zhboya.shinyapps.io/Interactive/> enables an individual to calculate their personalized hazard ratio as a function of their own age, gender, and personal physiological factors, and compare their hazard ratio to the average male and female of the same age group. For example, a 60 years old smoking male with a GFR of 80, a white blood cell count of 3.5, and a plasma glucose level of 90 will return a hazard ratio of 1.71 which is compared to the median reference 60 years old male.

**
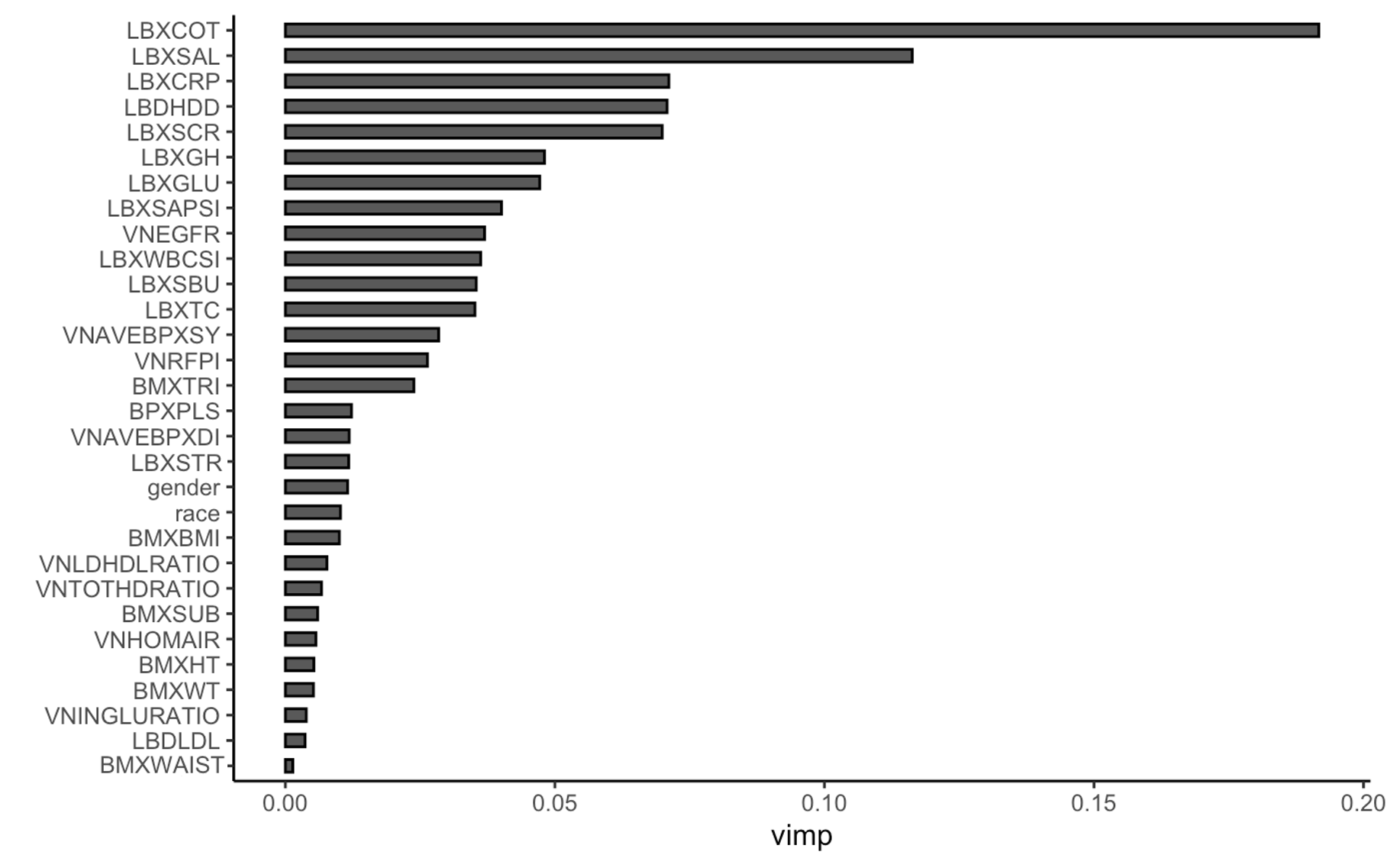
**

# Fig. S1. The VIMP ranking results for the RSF model using all 28 physiological indicators and 2 demographic indicators with age as the time scale.


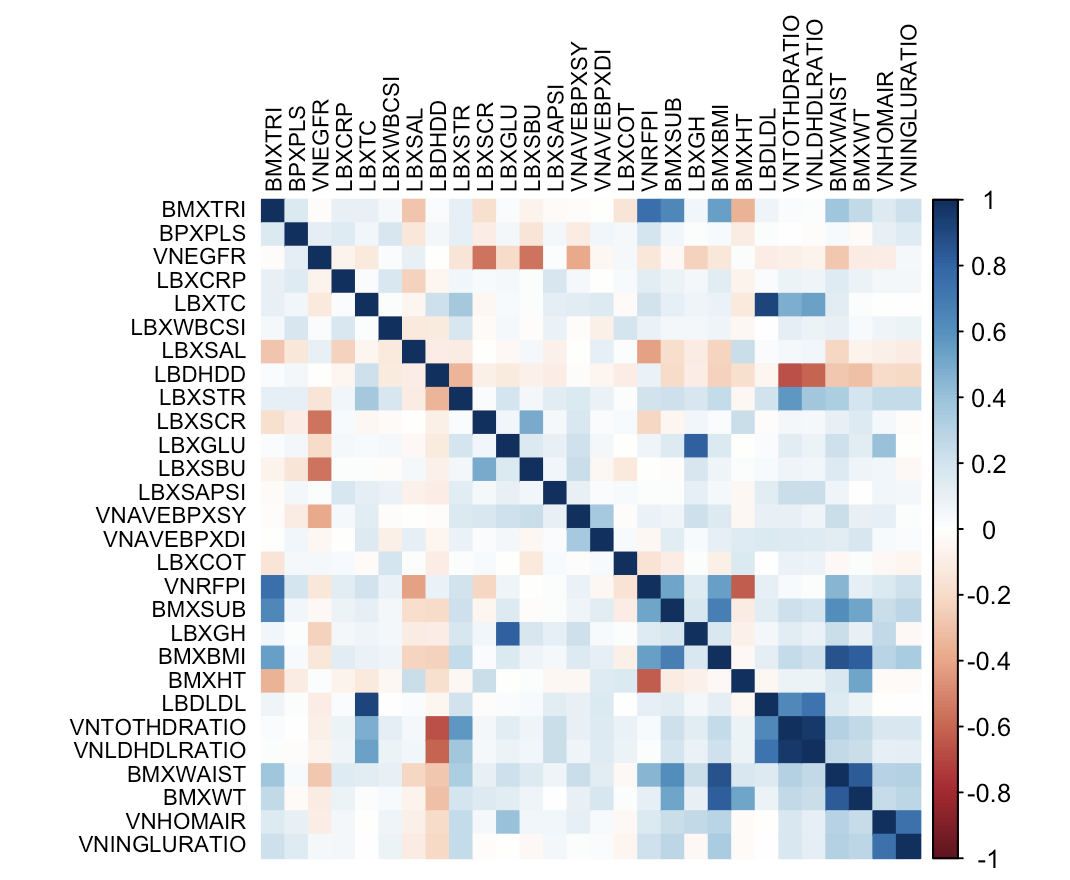


# Fig. S2. The correlation between different physiological indicato


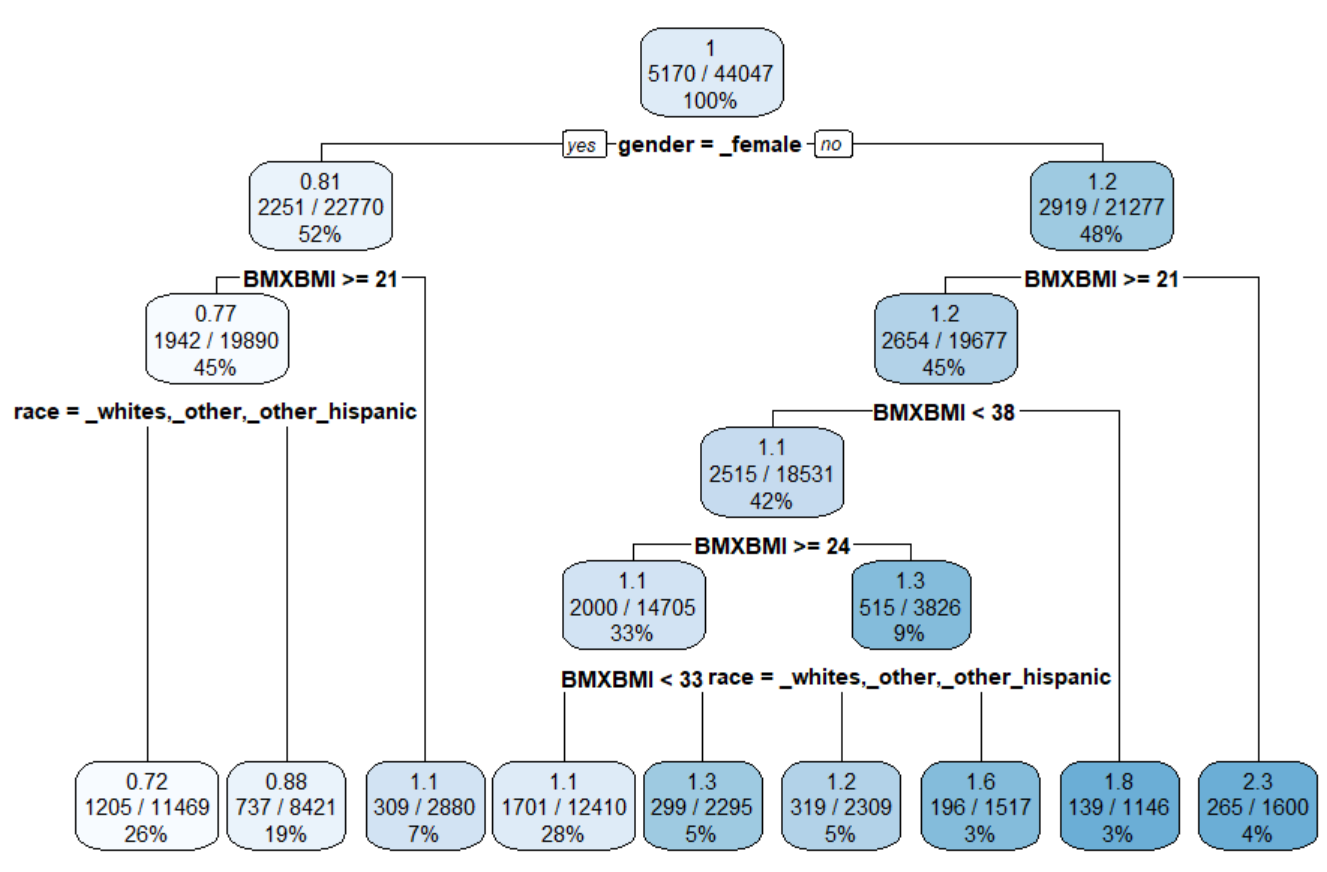


# Fig. S3. The survival tree structure and the identified risk groups for Body Mass Index (BMXBMI, kg/m**2). In each tree, three statistics are presented on the node which from top to bottom are: the relative risk, events/sample size, and the proportion of this group to the total population. At each split, the left branch is the group that meets the criterion shown under the node. The concordance for this survival tree model is 0.5830473. The complexity parameter selected in this case is 0.0001.


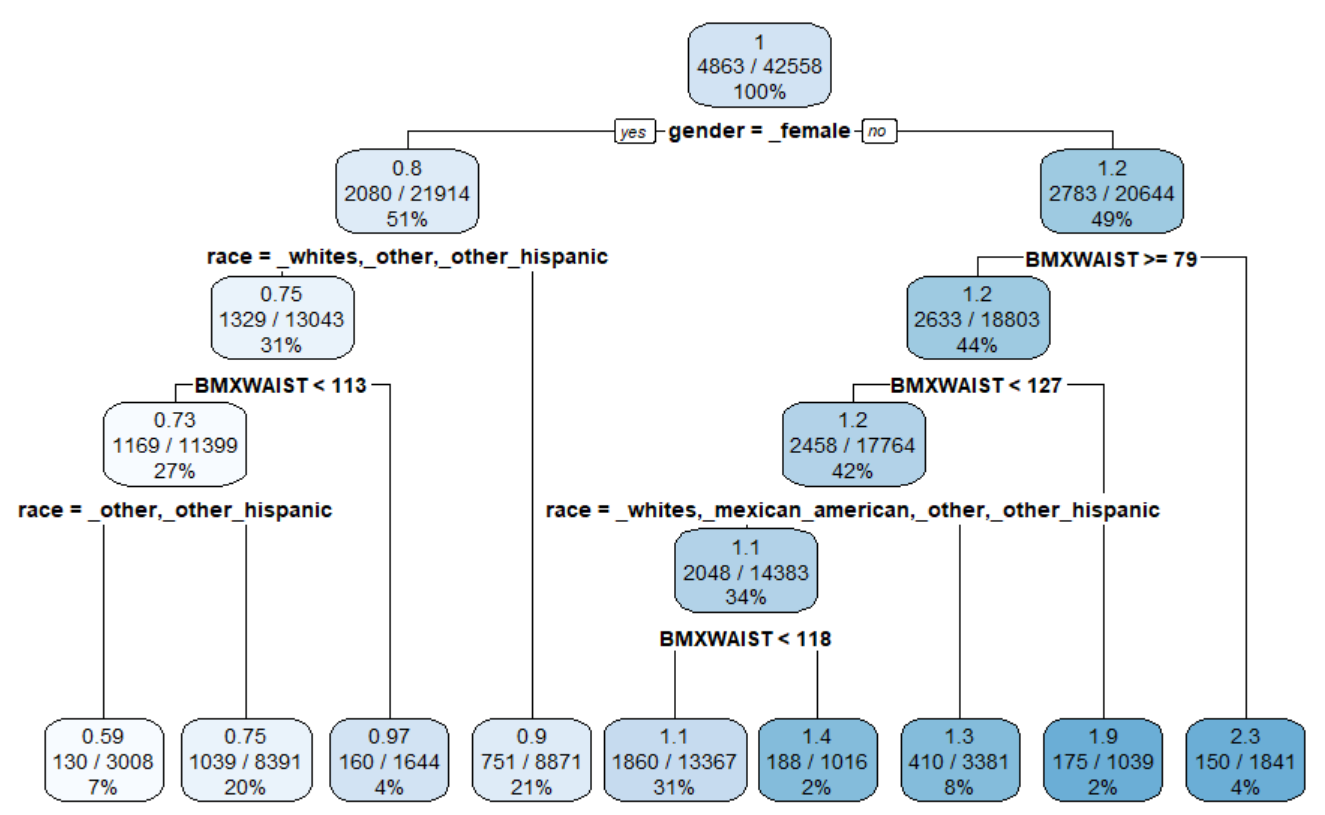


# Fig. S4. The survival tree structure and the identified risk groups for Waist Circumference (BMXWAIST, cm). The concordance for this survival tree model is 0.5814996. The complexity parameter selected in this case is 0.0003.


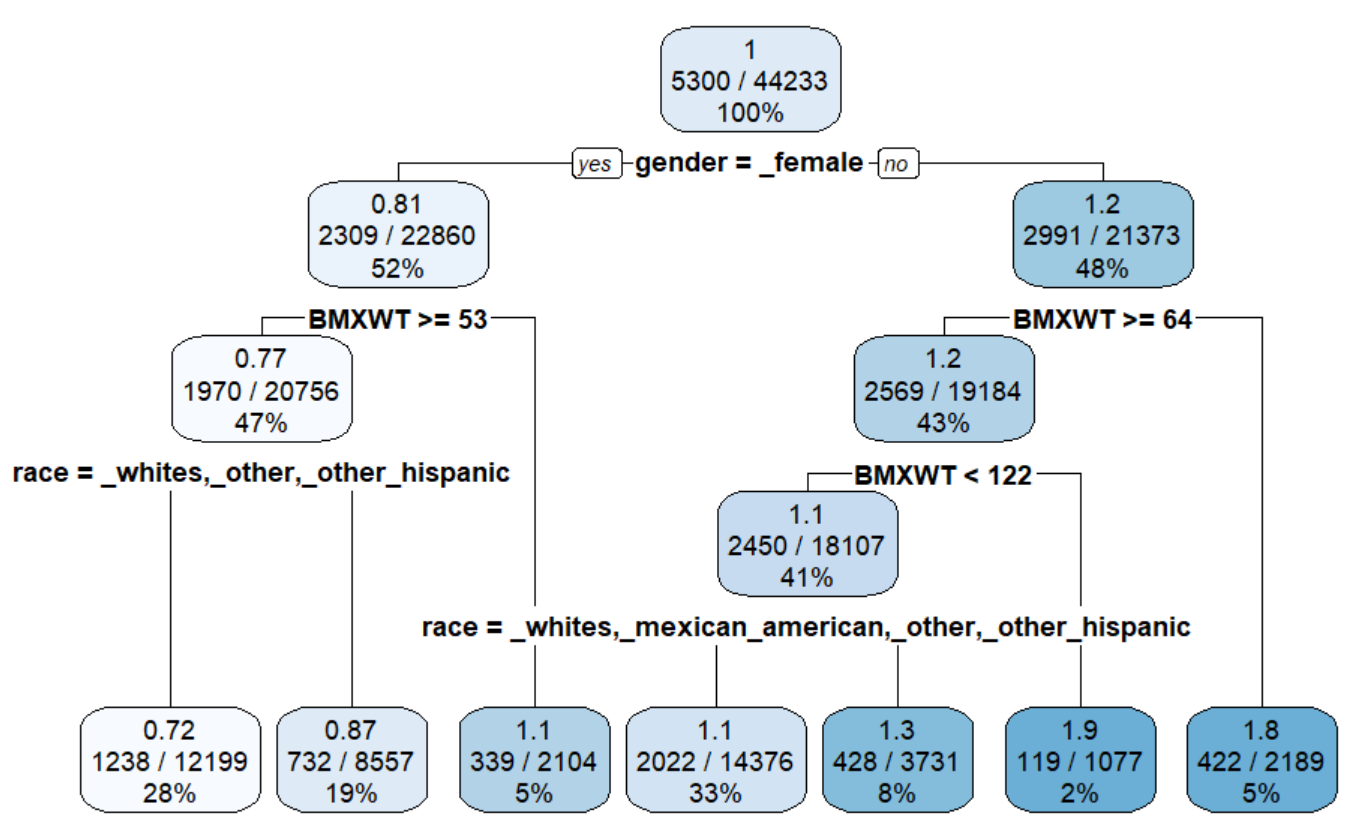


# Fig. S5. The survival tree structure and the identified risk groups for Weight (BMXWT, kg). The concordance for this survival tree model is 0.5809345. The complexity parameter selected in this case is 0.0003.


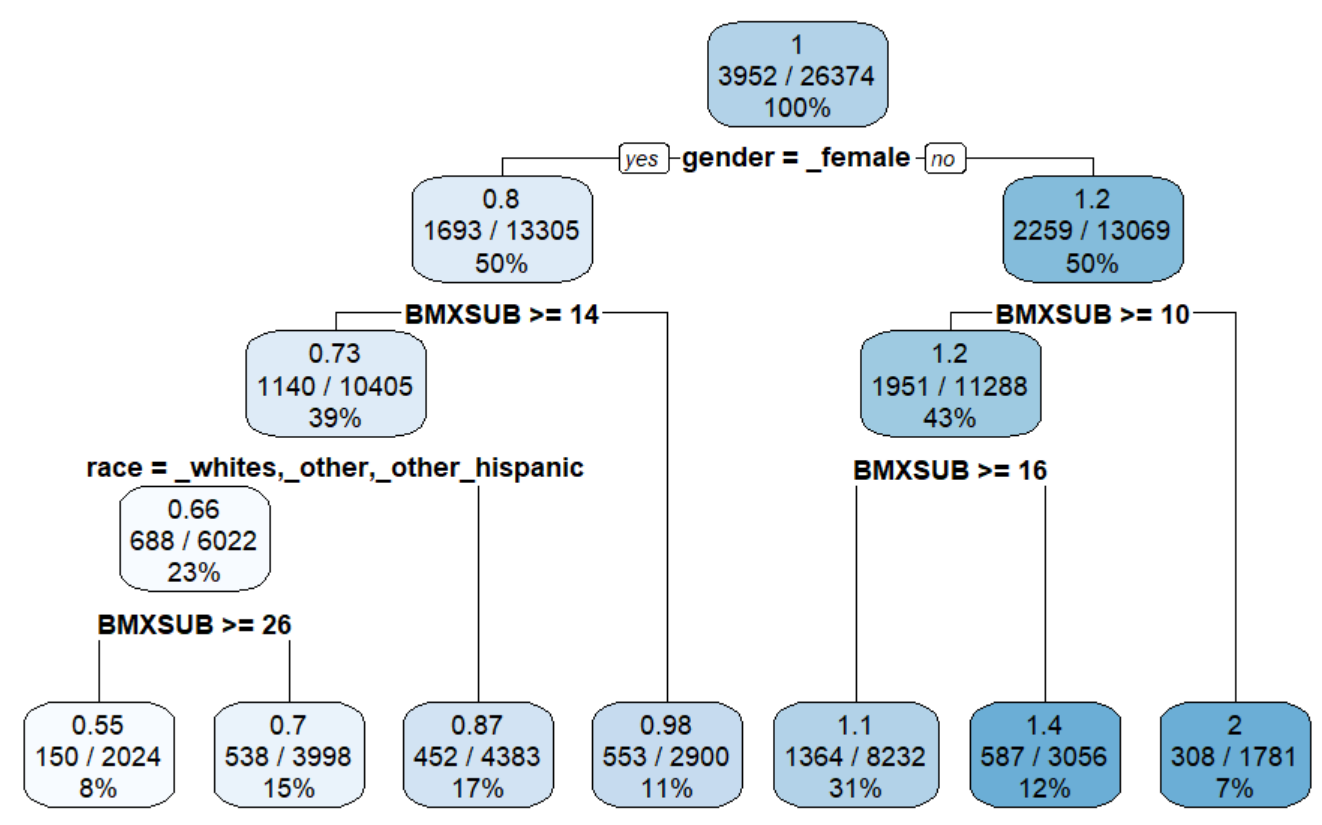


# Fig. S6. The survival tree structure and the identified risk groups for Subscapular Skinfold (BMXSUB, mm). The concordance for this survival tree model is 0.5879075. The complexity parameter selected in this case is 0.0003.


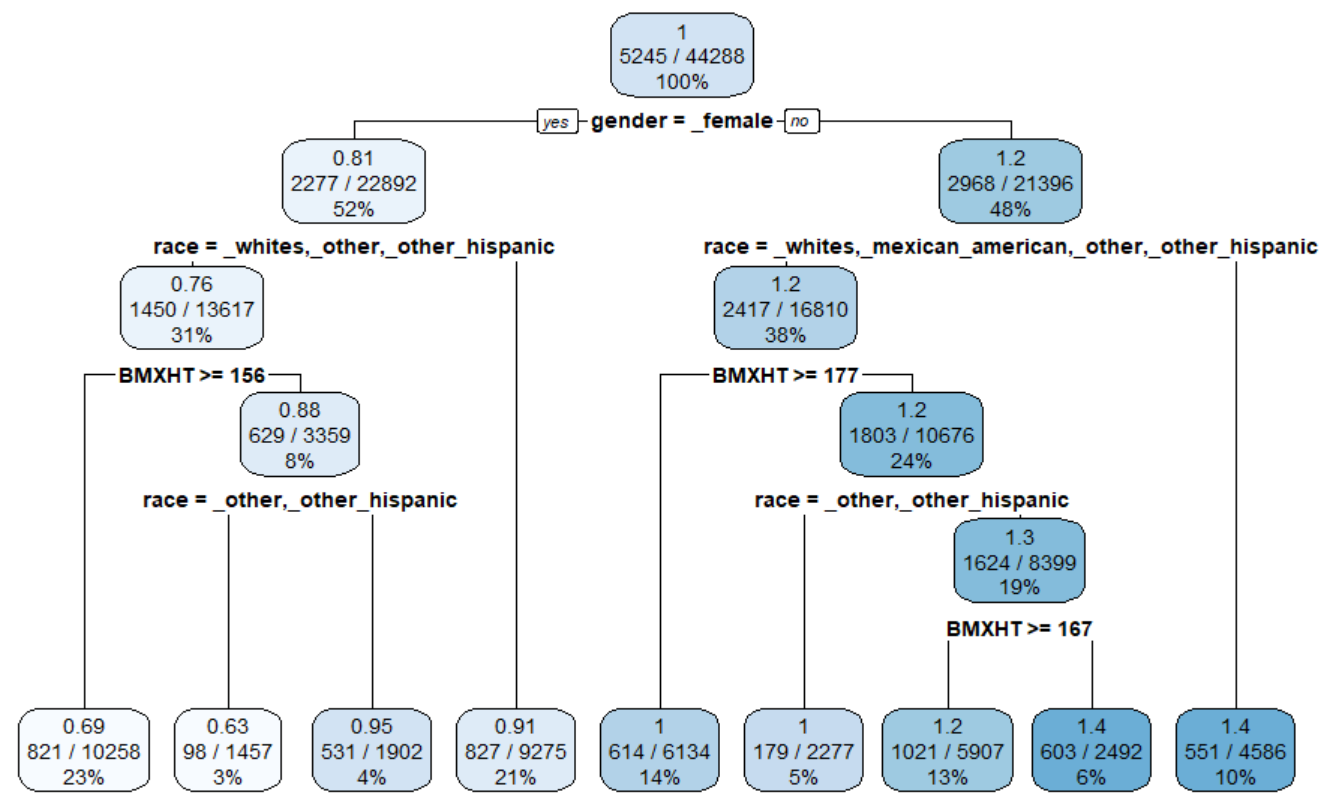


# Fig. S7. The survival tree structure and the identified risk groups for Standing Height (BMXHT, cm). The concordance for this survival tree model is 0.5790246. The complexity parameter selected in this case is 0.0003.


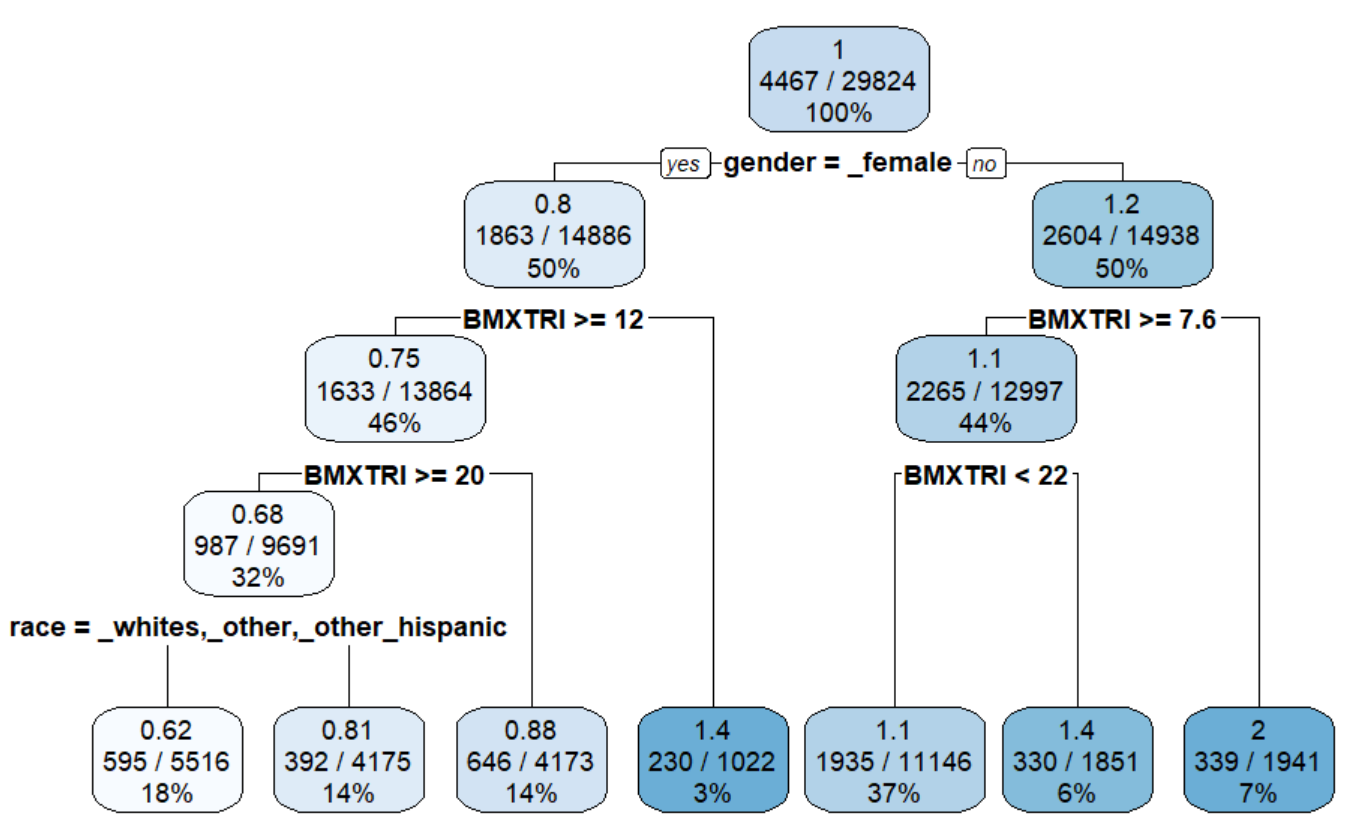


# Fig. S8. The survival tree structure and the identified risk groups for Triceps Skinfold (BMXTRI, mm). The concordance for this survival tree model is 0.5904533. The complexity parameter selected in this case is 0.0005.


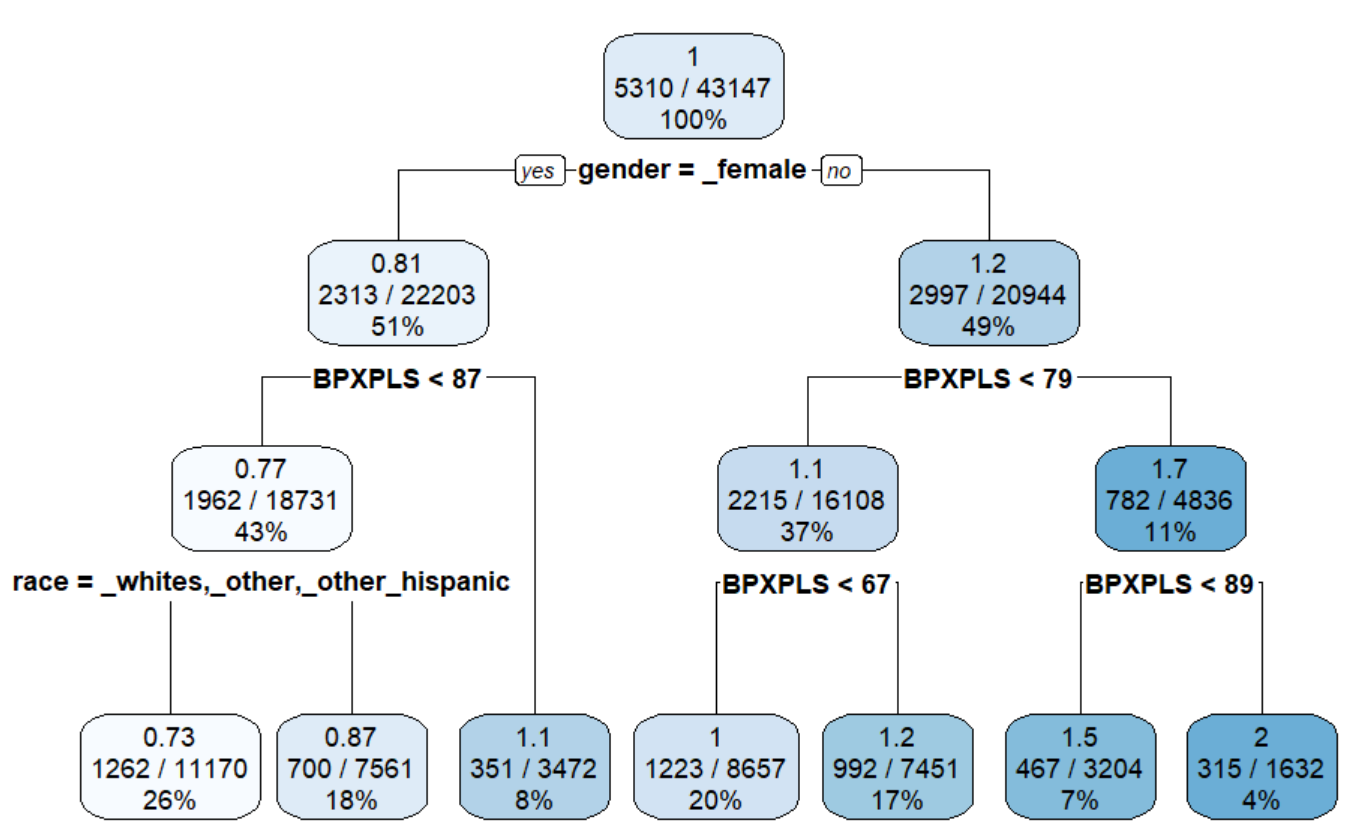


# Fig. S9. The survival tree structure and the identified risk groups for 60 sec. pulse (BPXPLS). The concordance for this survival tree model is 0.5863917. The complexity parameter selected in this case is 0.0005.


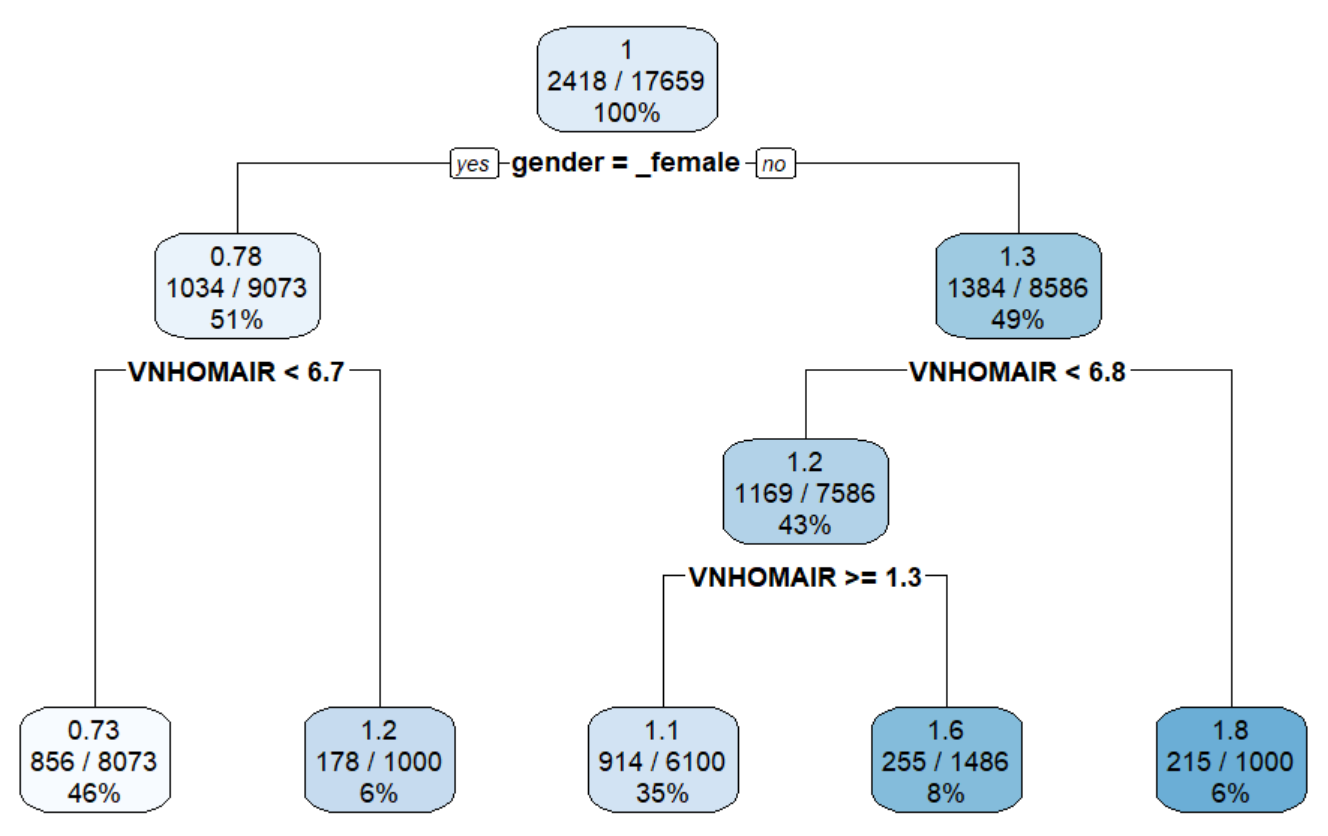


# Fig. S10. The survival tree structure and the identified risk groups for Homeostatic Model Assessment of Insulin Resistance (VNHOMAIR).

The concordance for this survival tree model is 0.5913751. The complexity parameter selected in this case is 0.001.


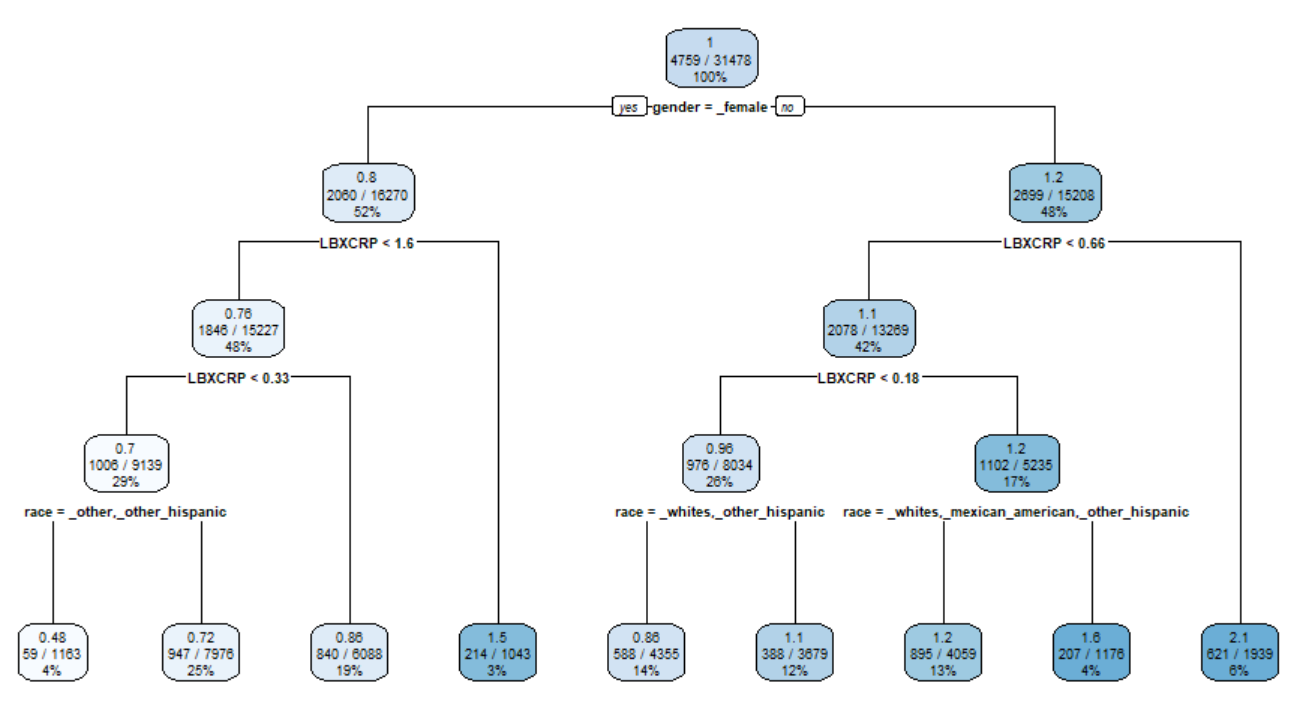


# Fig. S11. The survival tree structure and the identified risk groups for C-reactive protein (LBXCRP, mg/dL). The concordance for this survival tree model is 0.6039363. The complexity parameter selected in this case is 0.0005.


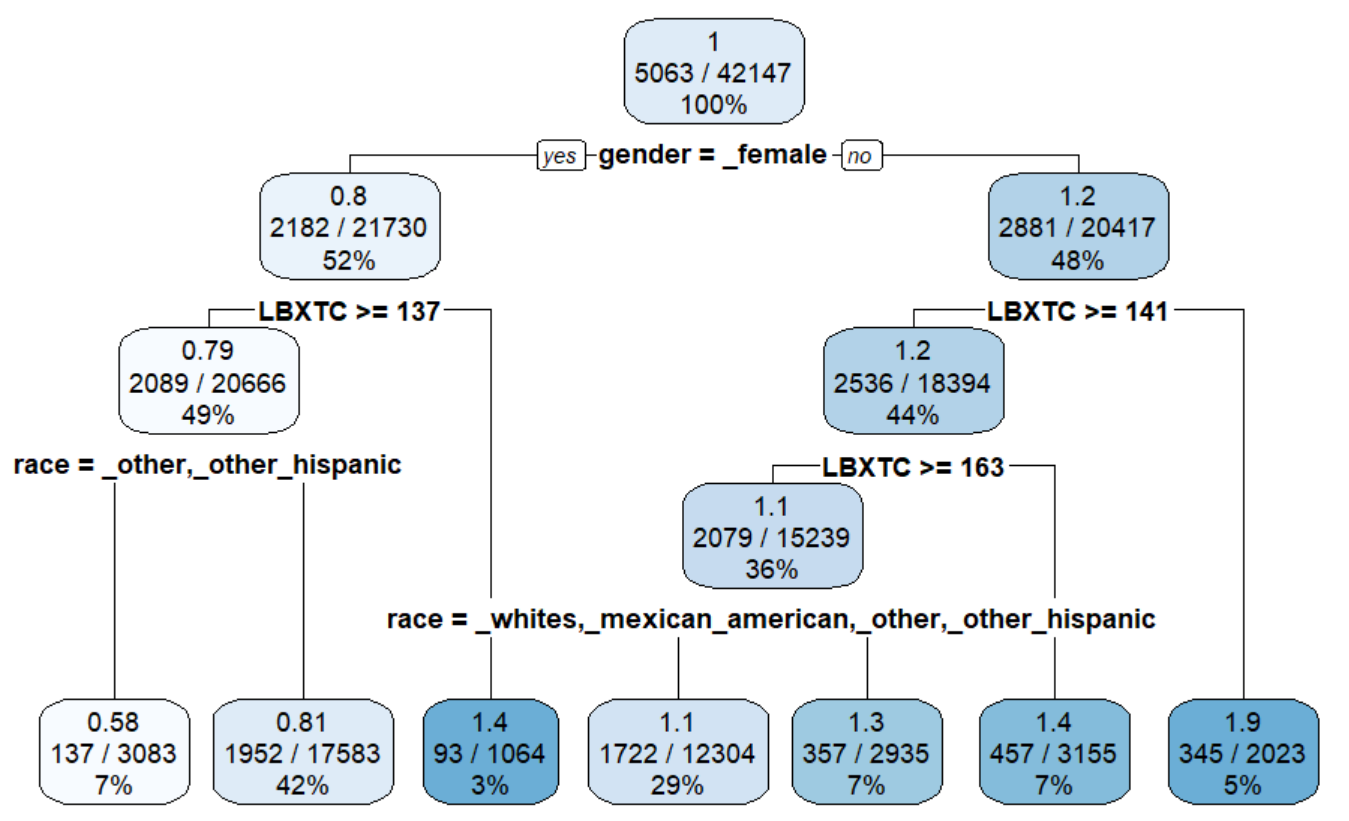


# Fig. S12. The survival tree structure and the identified risk groups for Total cholesterol (LBXTC, mg/dL).The concordance for this survival tree model is 0.5760108. The complexity parameter selected in this case is 0.0005.


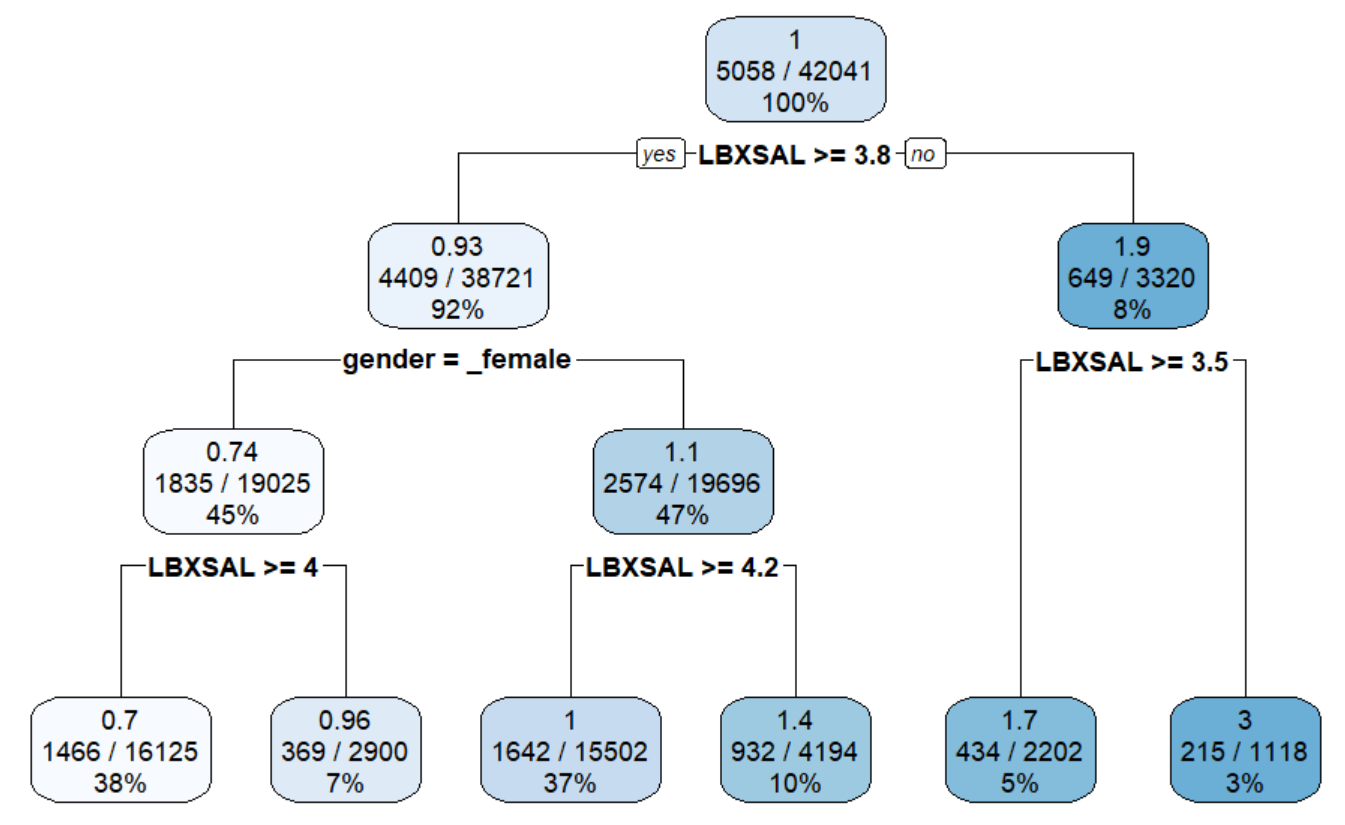


# Fig. S13. The survival tree structure and the identified risk groups for T Albumin (LBXSAL, g/dL). The concordance for this survival tree model is 0.5975818. The complexity parameter selected in this case is 0.001.


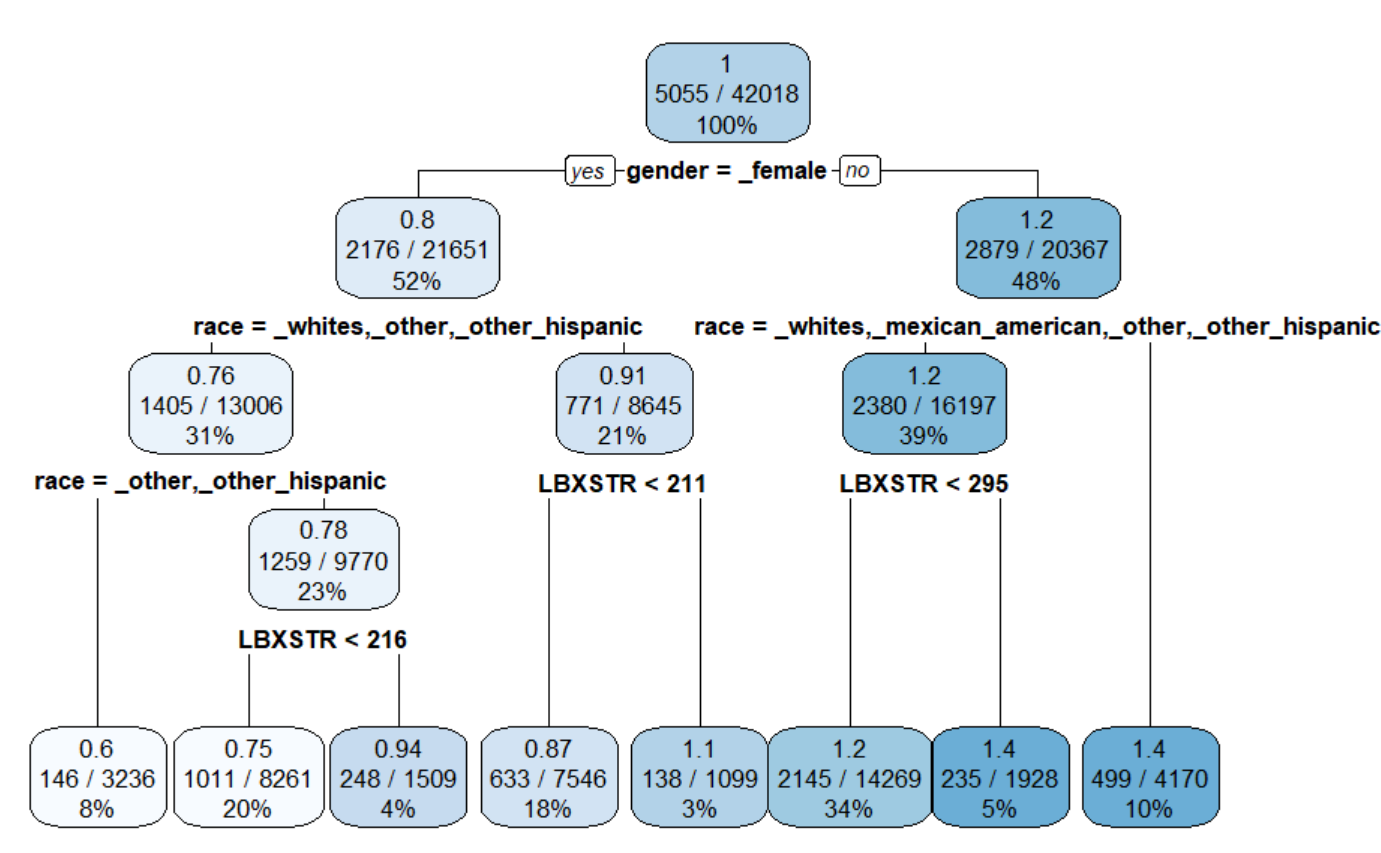


# Fig. S14. The survival tree structure and the identified risk groups for Triglycerides (LBXSTR, mg/dL). The concordance for this survival tree model is 0.5743658. The complexity parameter selected in this case is 0.0003.


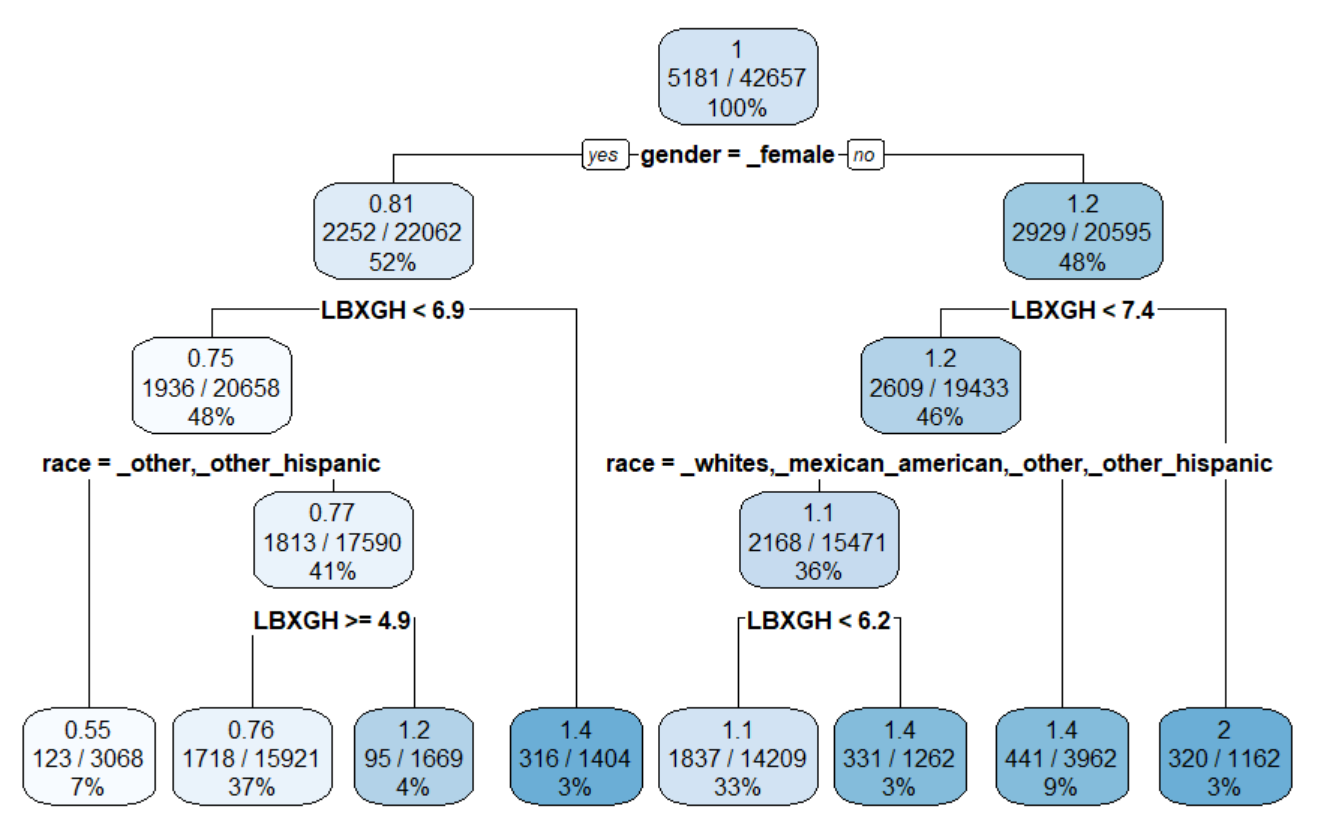


# Fig. S15. The survival tree structure and the identified risk groups for Glycohemoglobin (LBXGH, %). The concordance for this survival tree model is 0.5860696. The complexity parameter selected in this case is 0.0005.


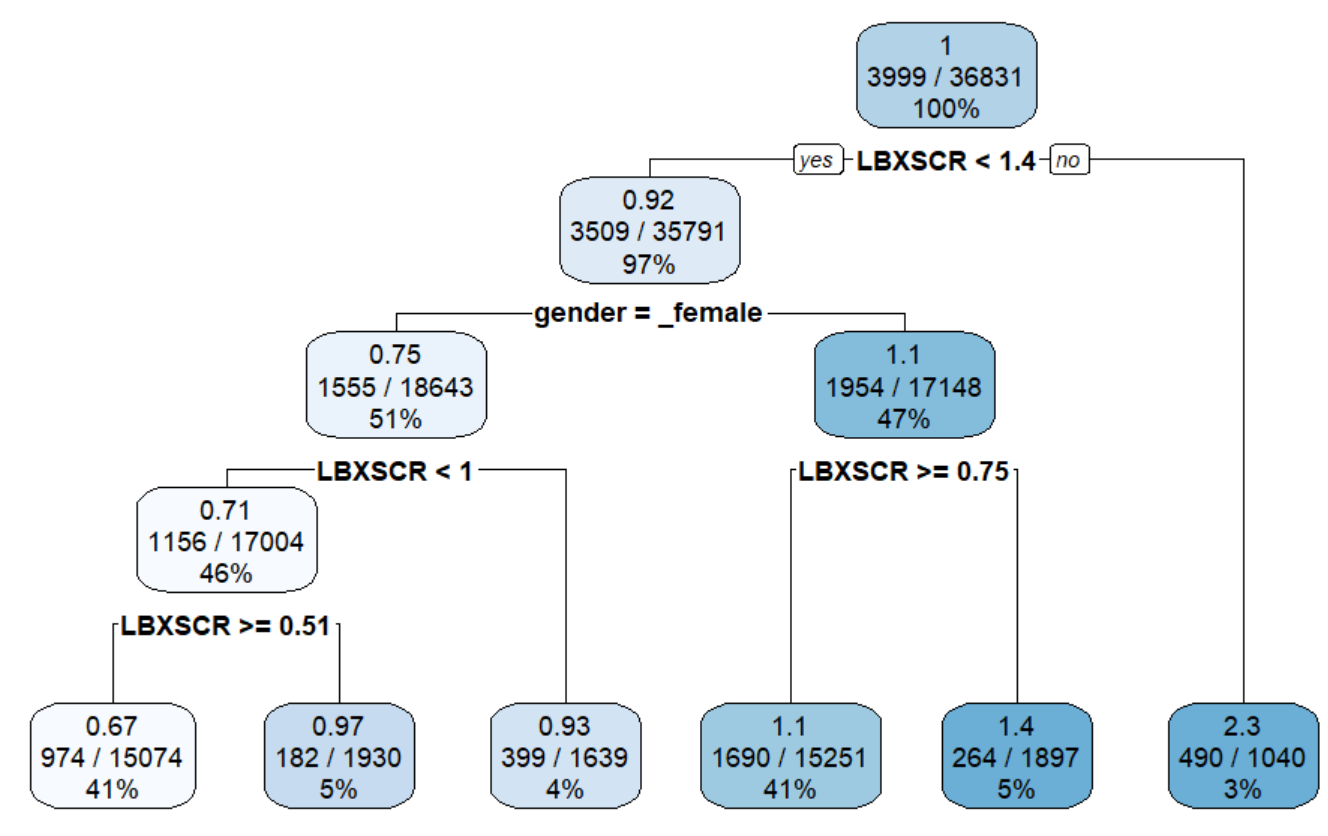


# Fig. S16. The survival tree structure and the identified risk groups for Creatinine (LBXSCR, mg/dL). The concordance for this survival tree model is 0.5970776. The complexity parameter selected in this case is 0.0005.


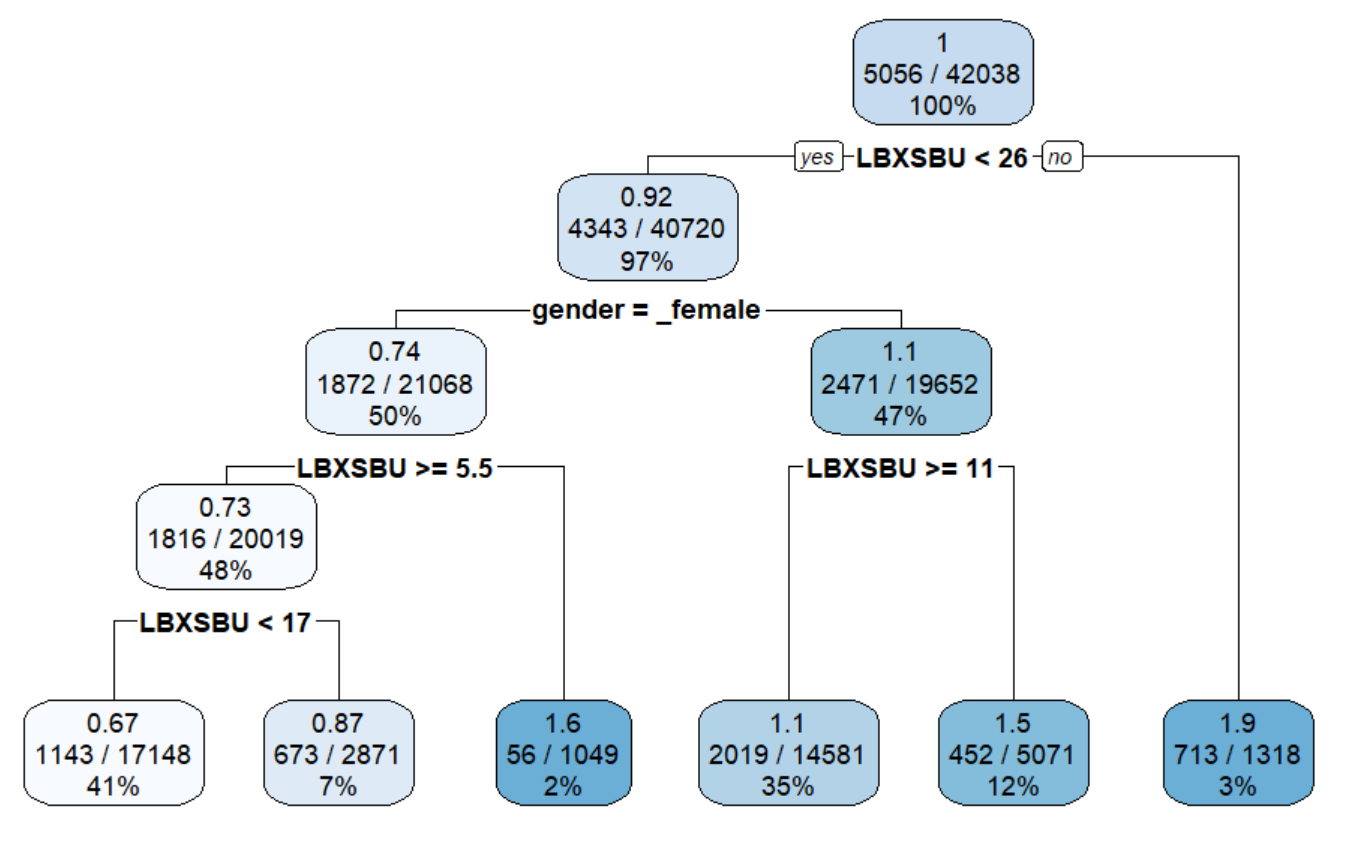


# Fig. S17. The survival tree structure and the identified risk groups for Blood urea nitrogen (LBXSBU, mg/dL). The concordance for this survival tree model is 0.59893. The complexity parameter selected in this case is 0.0005.


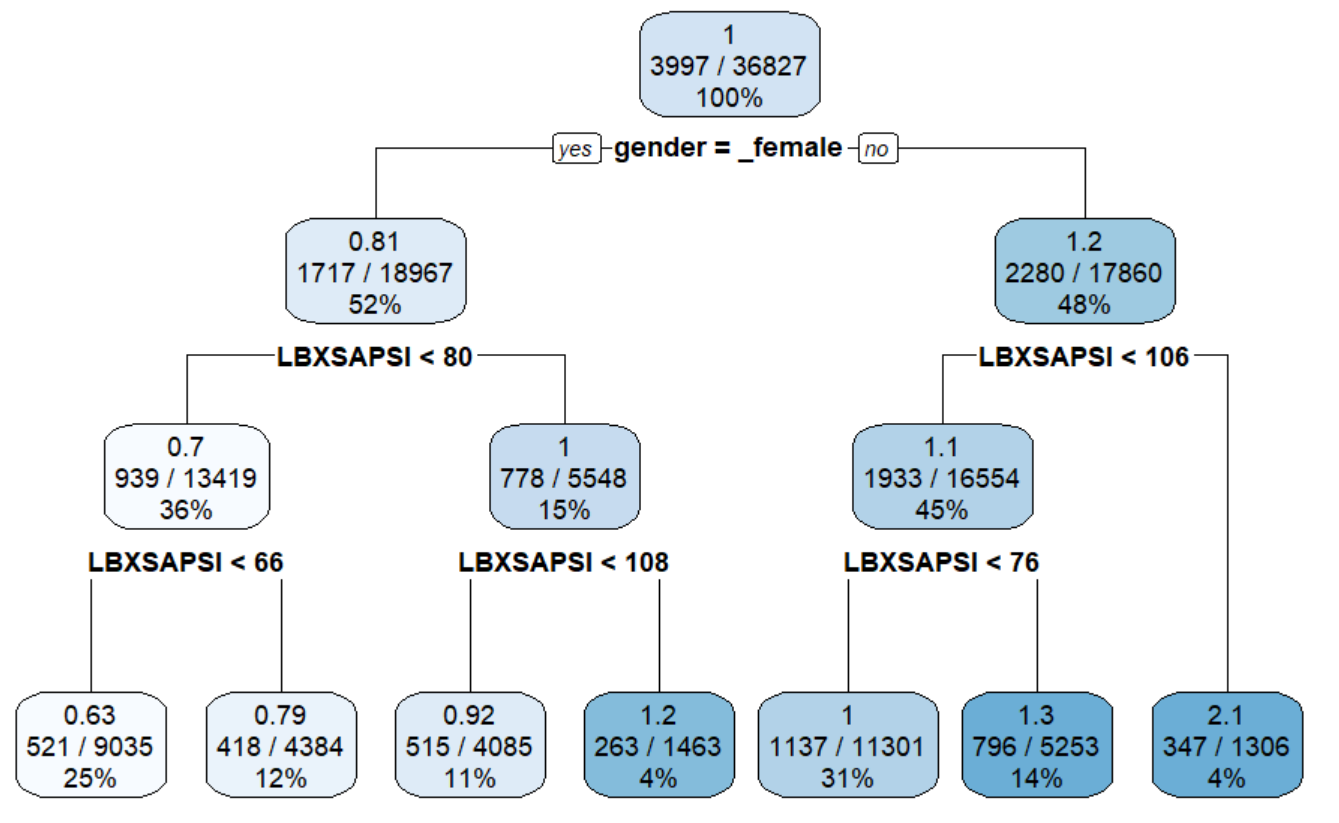


# Fig. S18. The survival tree structure and the identified risk groups for Alkaline phosphatase (LBXSAPSI, U/L). The concordance for this survival tree model is 0.5944562. The complexity parameter selected in this case is 0.0005.


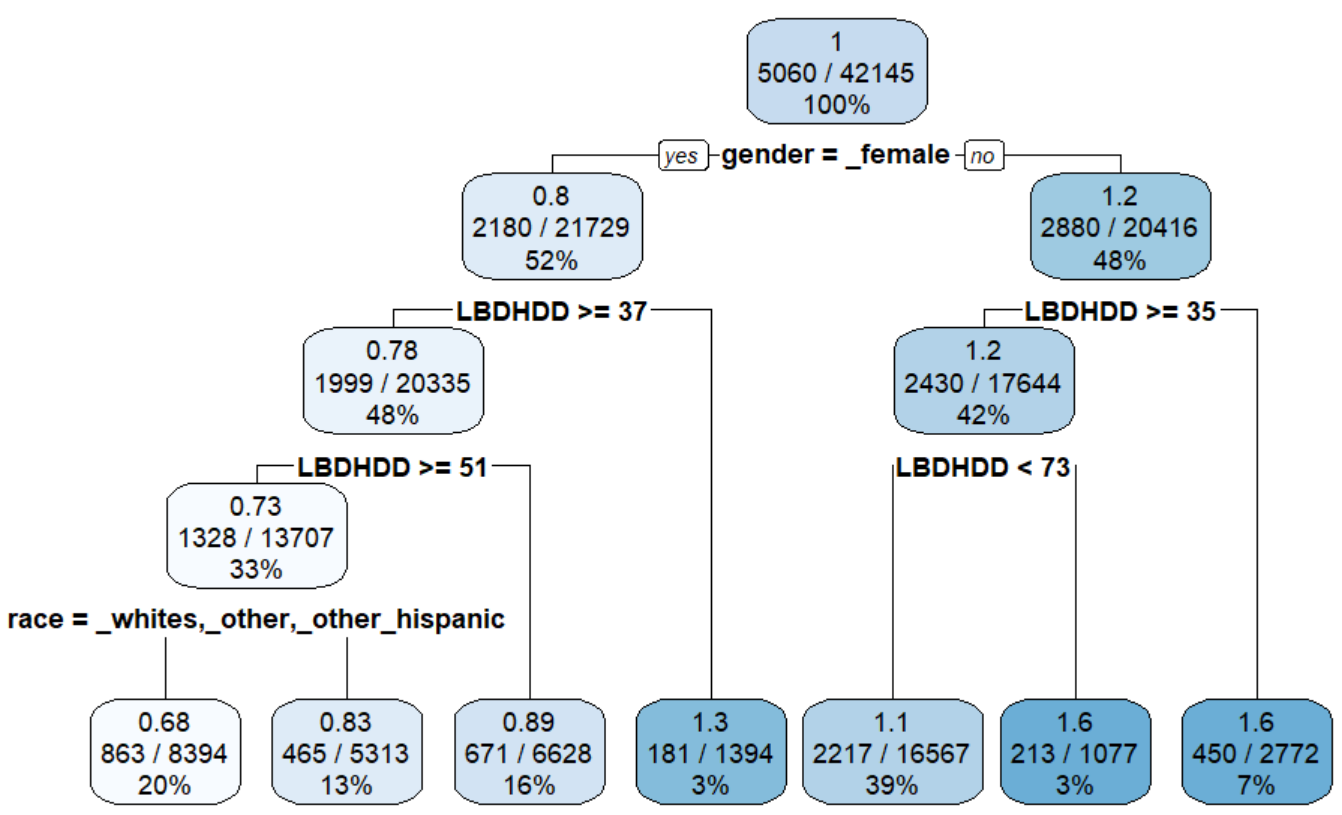


# Fig. S19. The survival tree structure and the identified risk groups for Direct HDL-Cholesterol (LBDHDD, mg/dL). The concordance for this survival tree model is 0.5814263. The complexity parameter selected in this case is 0.0005.


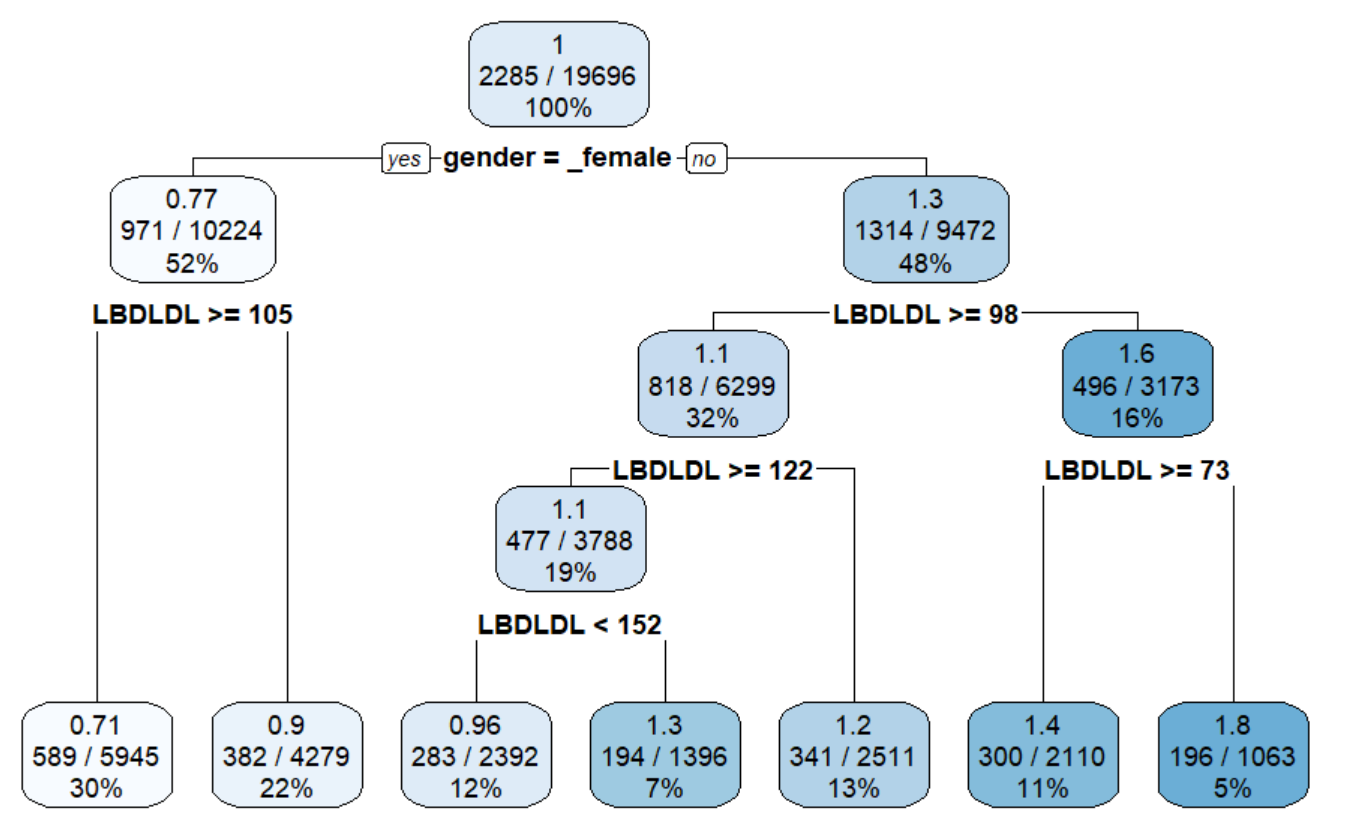


# Fig. S20. The survival tree structure and the identified risk groups for LDL-cholesterol (LBDLDL, mg/dL). The concordance for this survival tree model is 0.5900448. The complexity parameter selected in this case is 0.0005.


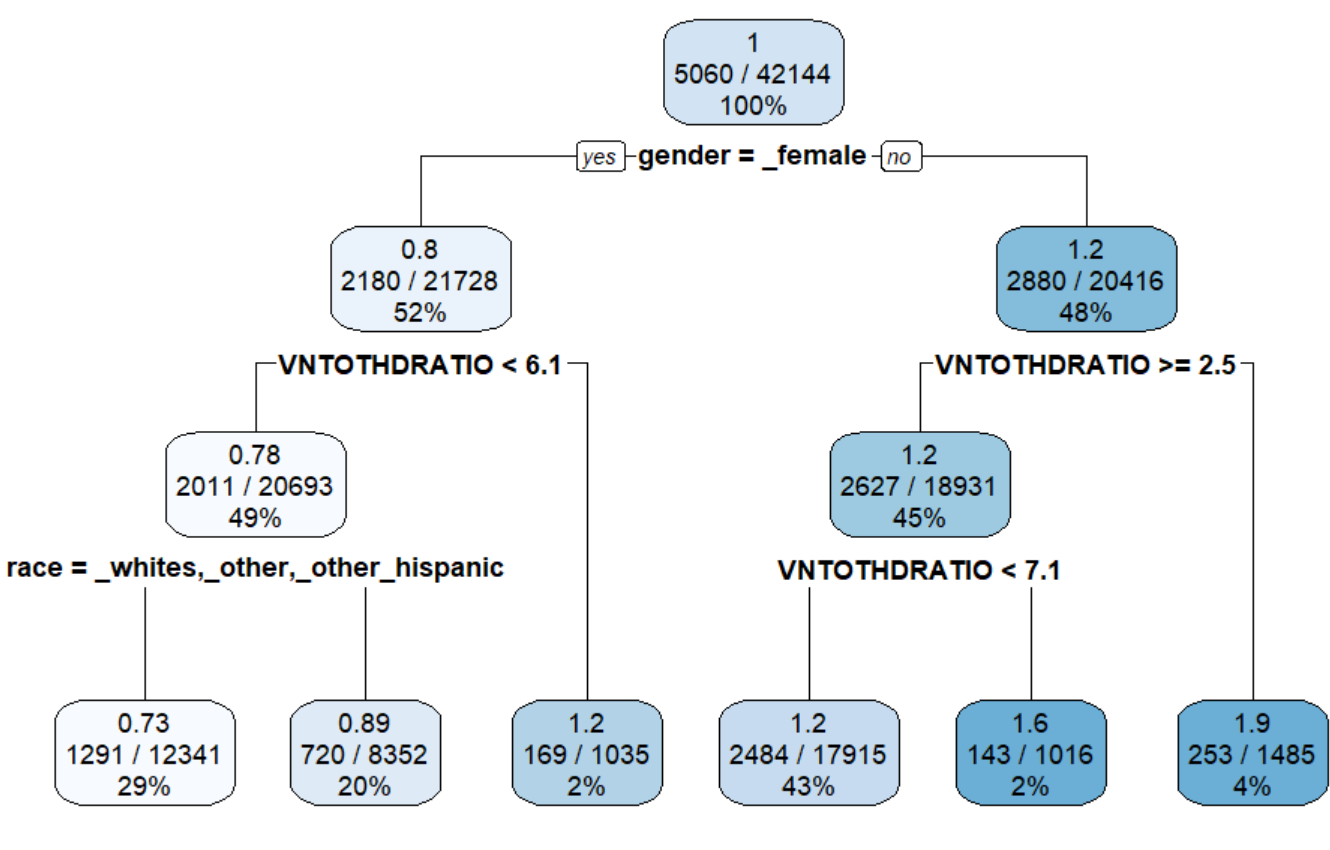


# Fig. S21. The survival tree structure and the identified risk groups for Ratio of Total to HDL Cholesterol (VNTOTHDRATIO). The concordance for this survival tree model is 0.5750106. The complexity parameter selected in this case is 0.0005.


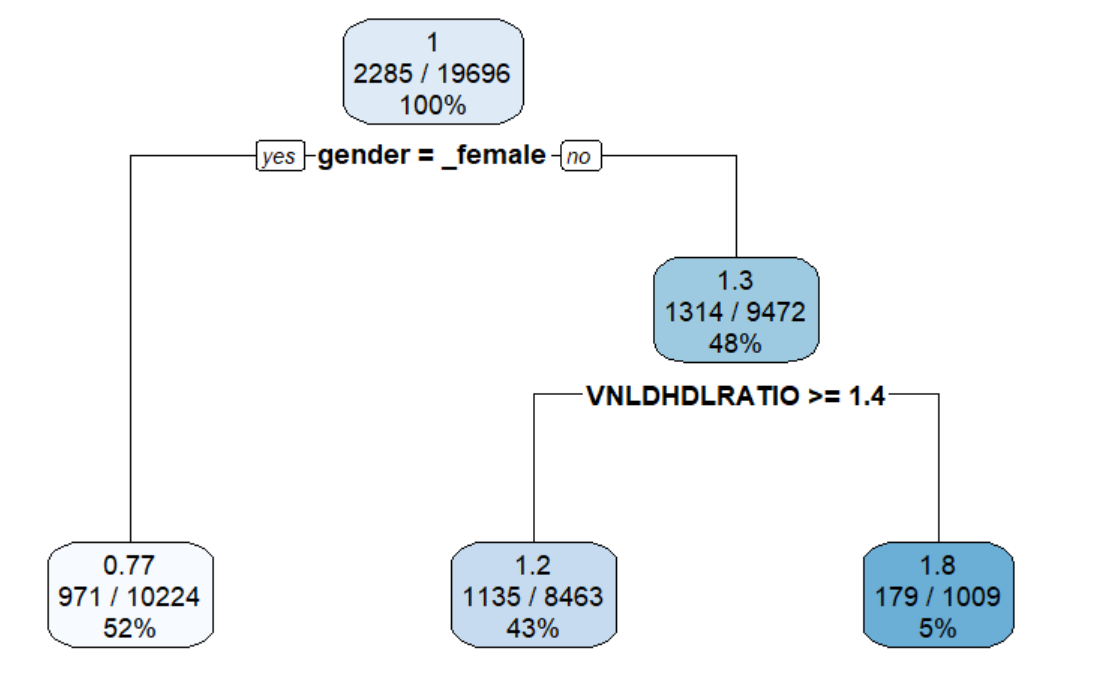


# Fig. S22. The survival tree structure and the identified risk groups for Ratio of LDL to HDL Cholesterol (VNLDHDLRATIO). The concordance for this survival tree model is 0.5725829. The complexity parameter selected in this case is 0.001.


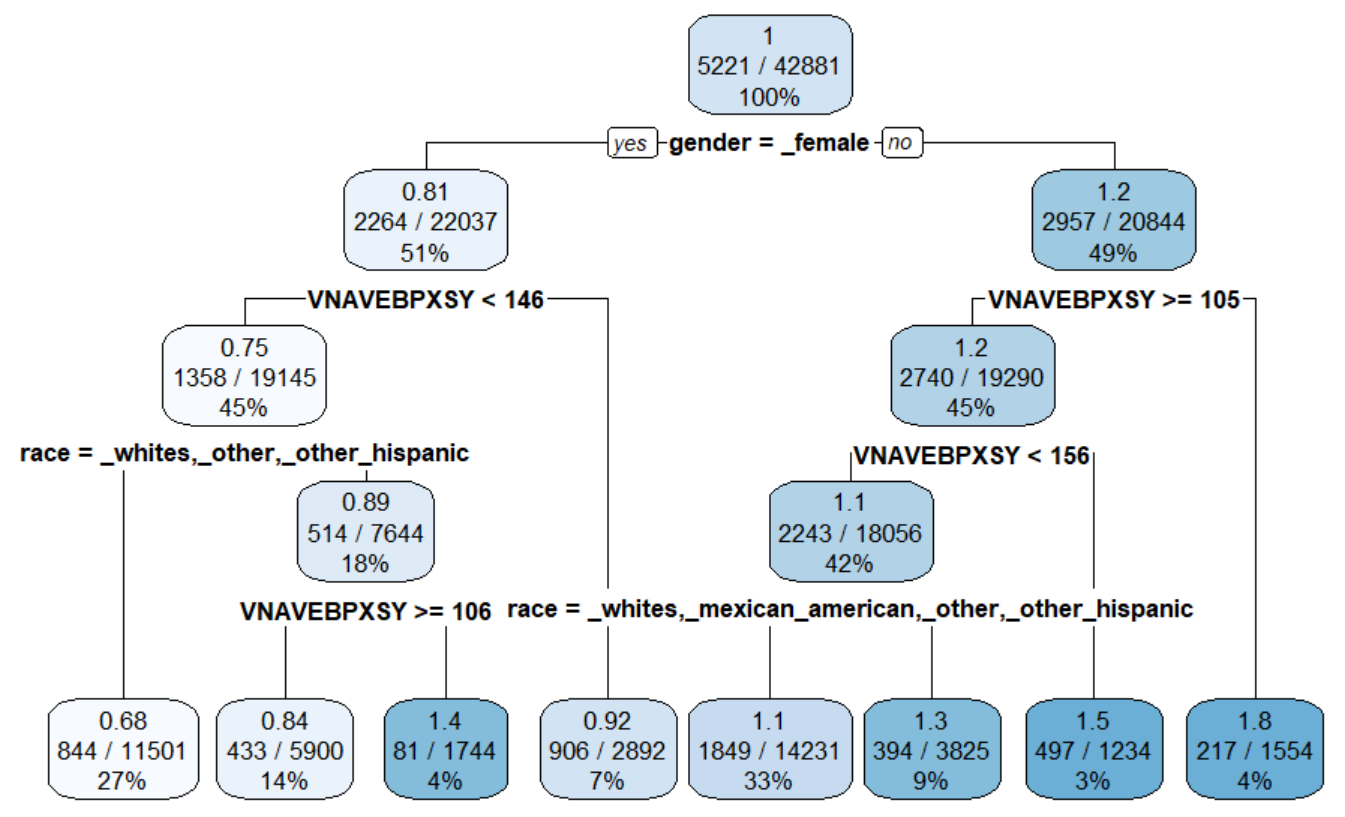


# Fig. S23. The survival tree structure and the identified risk groups for Systolic: Average blood pressure (VNAVEBPXSY, mm Hg). The concordance for this survival tree model is 0.5800699. The complexity parameter selected in this case is 0.0001.


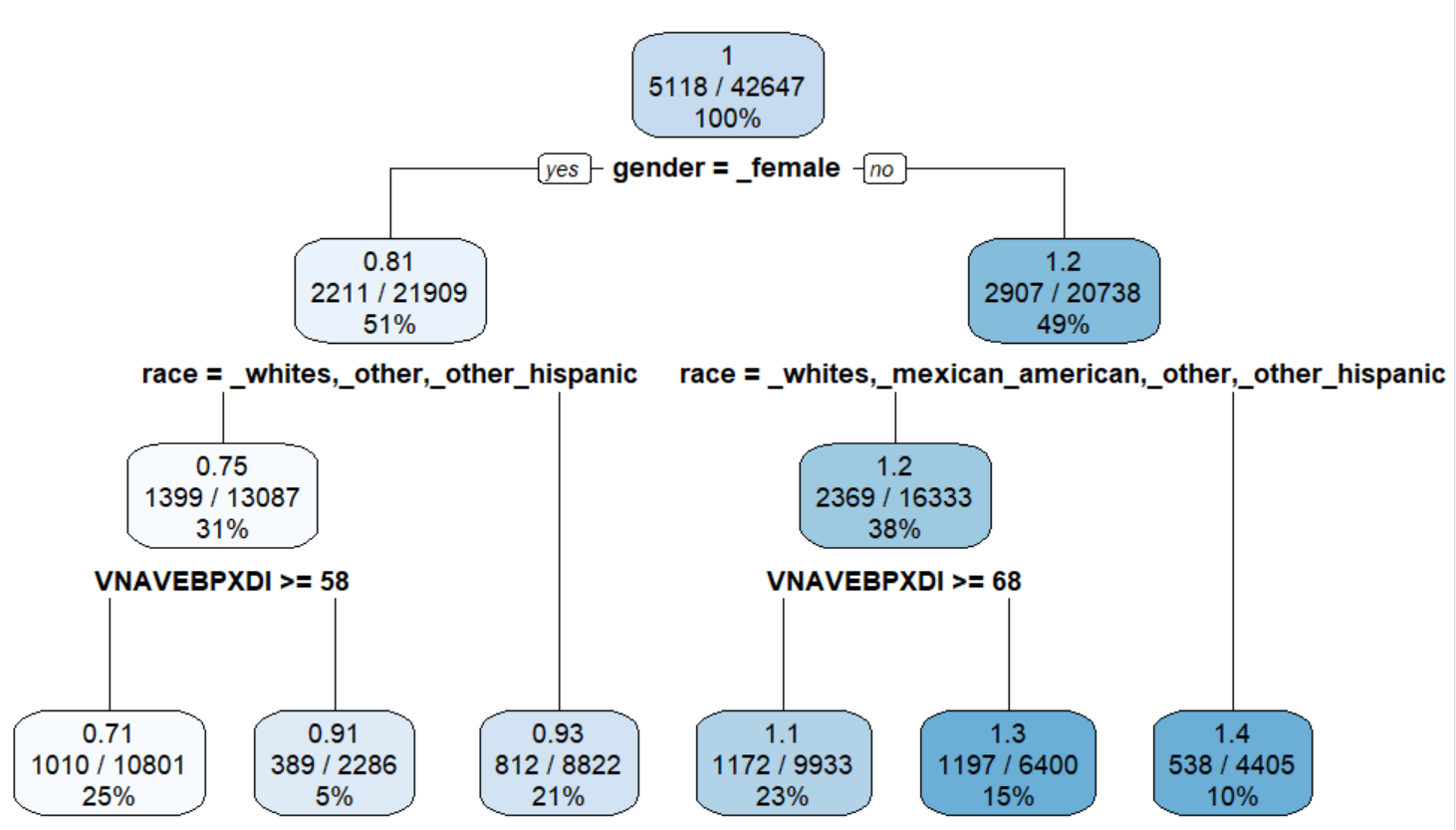


# Fig. S24. The survival tree structure and the identified risk groups for Diastolic: Average blood pressure (VNAVEBPXDI, mm Hg). The concordance for this survival tree model is 0.5759052. The complexity parameter selected in this case is 0.0001.


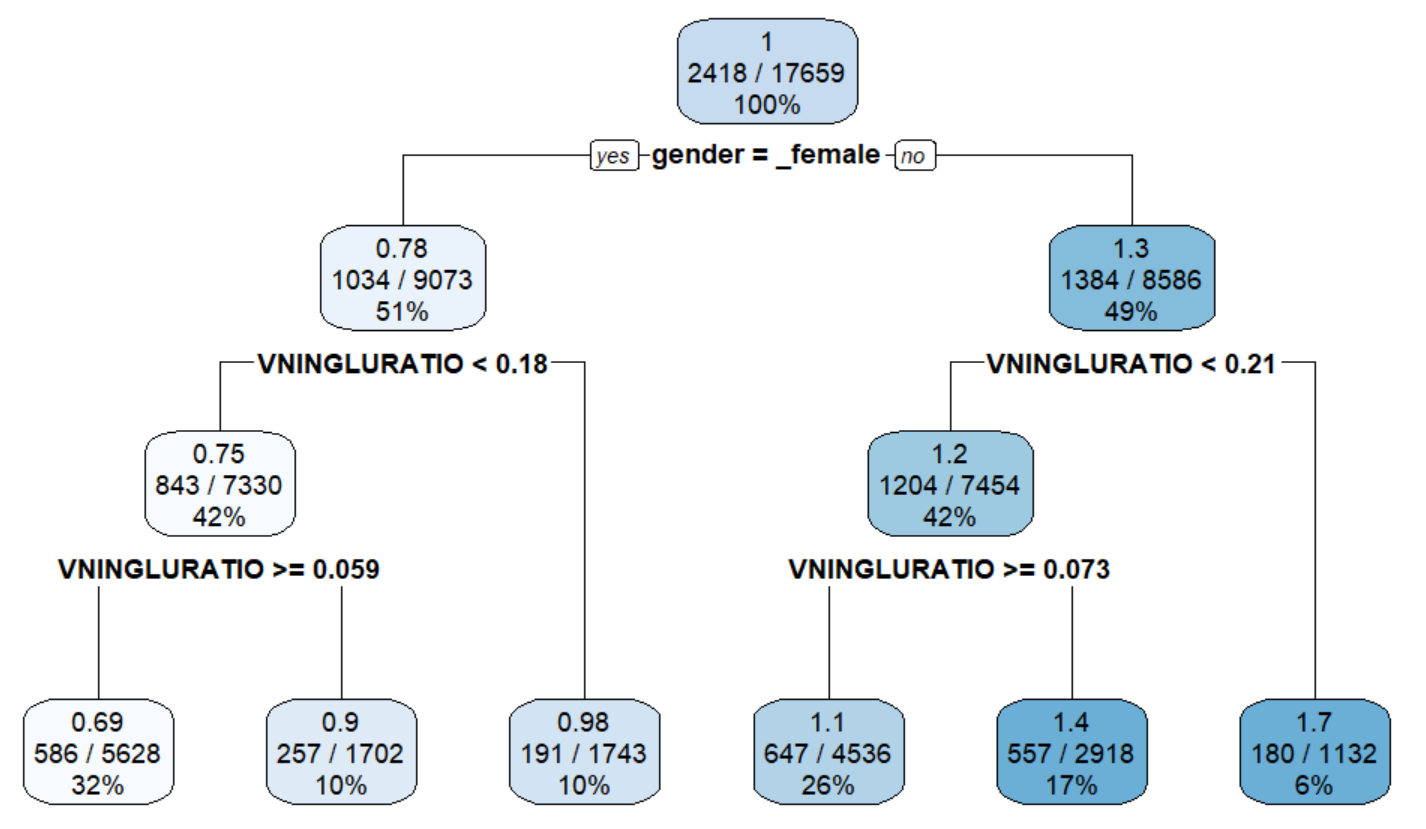


# Fig. S25. The survival tree structure and the identified risk groups for Ratio of Insulin to Glucose (VNINGLURATIO). The concordance for this survival tree model is 0.5894333. The complexity parameter selected in this case is 0.0001.


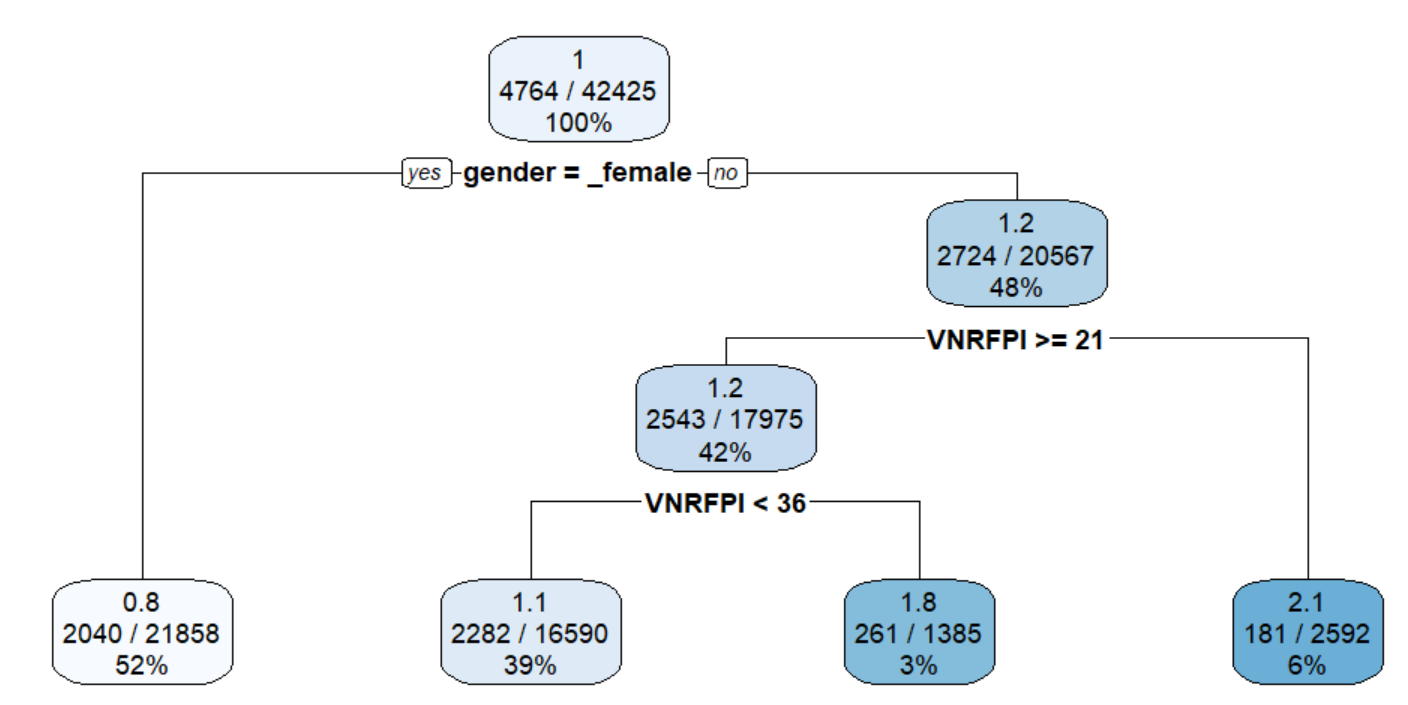


# Fig. S26. The survival tree structure and the identified risk groups for Relative Fat Mass Index (VNRFPI). The concordance for this survival tree model is 0.5887312. The complexity parameter selected in this case is 0.001.

# Fig. S27. The identified risk groups for a) serum cotinine, b) GFR, c) plasma glucose, d) white blood cell count, and e) all of these four factors together while adjusting for sex using survival tree models by using different sample sizes. Each node box presents three statistics, which from top to bottom are the relative risk, the events and sample sizes, and the proportion of this group to the total population.


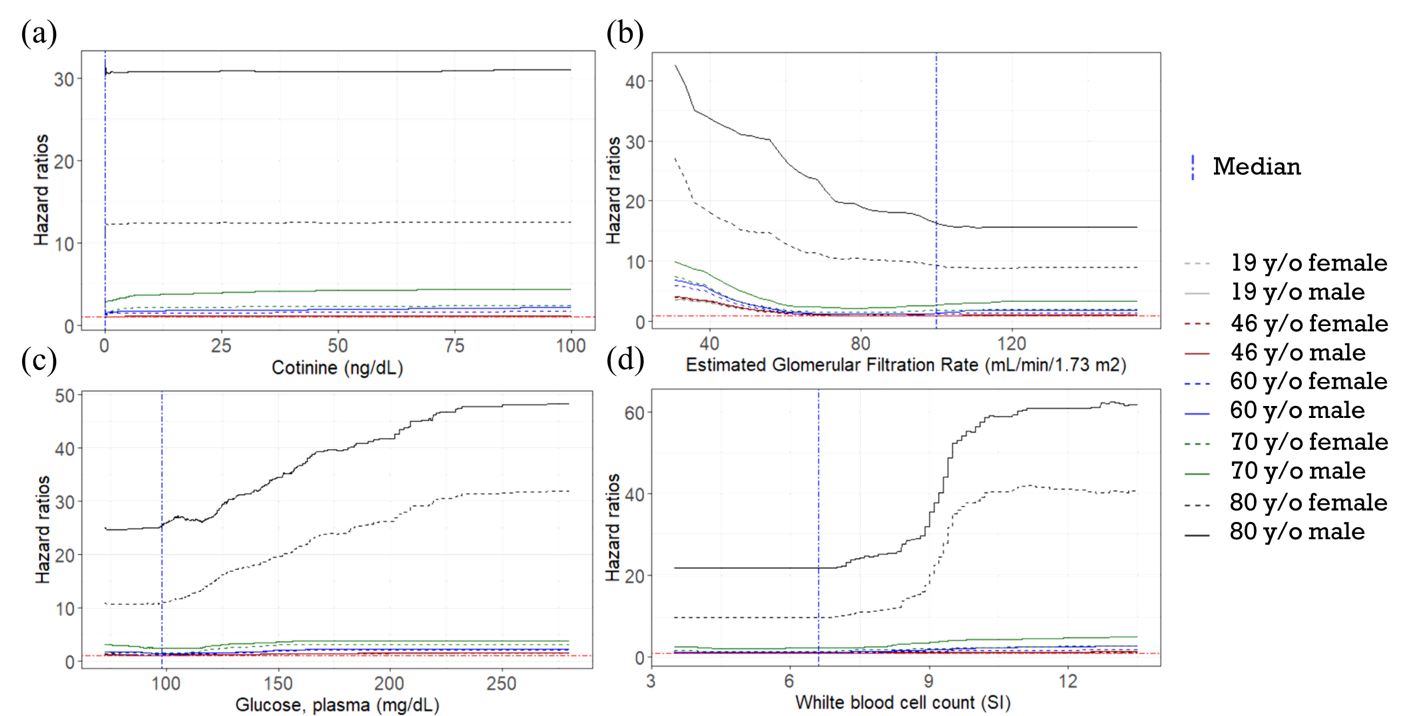


# Fig. S28. Predicted variation in HR as a function of each of the 4 indicators (cotinine, glomerular filtration rate, glucose plasma, and white blood cell count) for different age and gender groups. The blue vertical dashed line represents the median value of the population for each indicator.


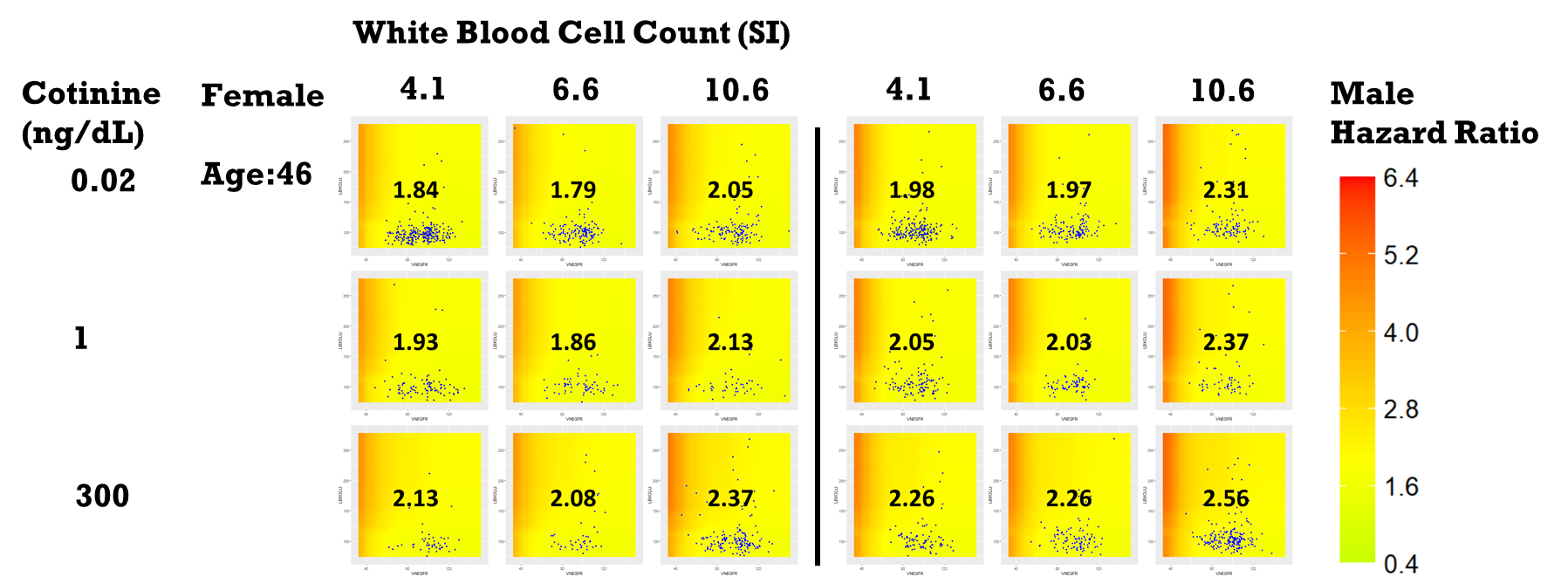


# Fig. S29. Combined effects of key physiological/demographic indicators for age groups of 46 (median age).


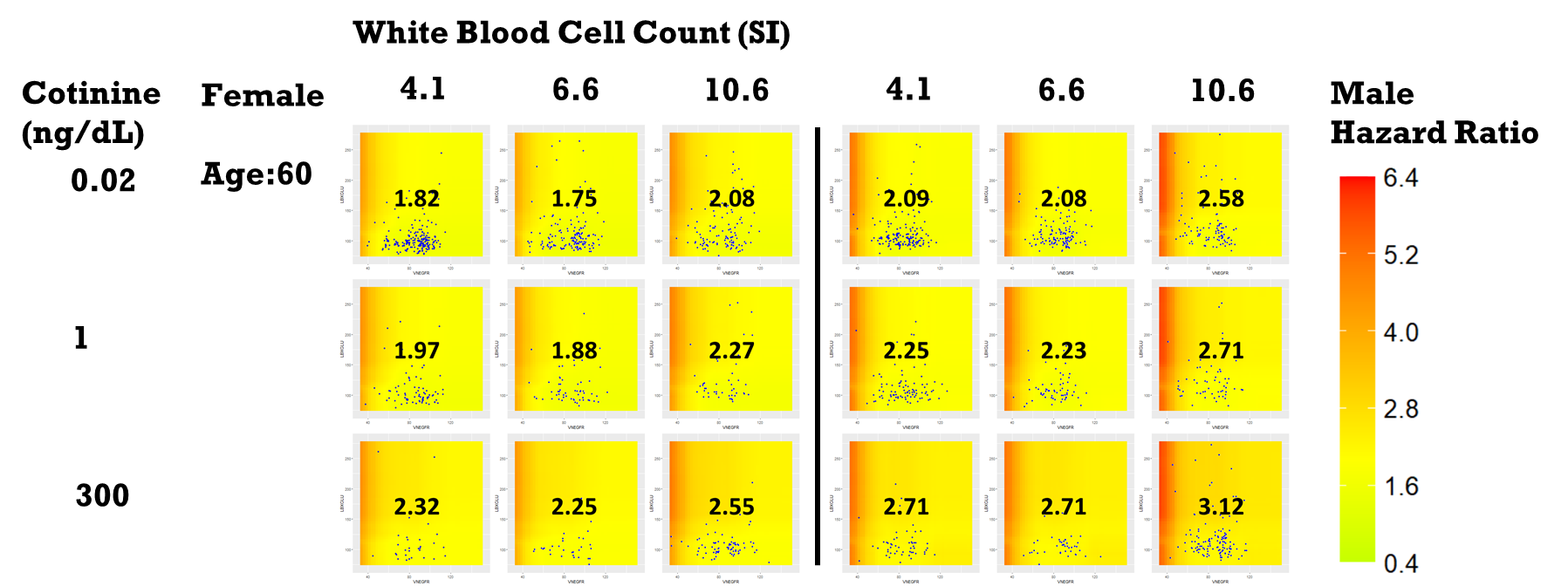


Fig. S30. The combined effects of key physiological/demographic indicators for age groups of 60.

Fig. S31. Workflow for data processing and model construction.


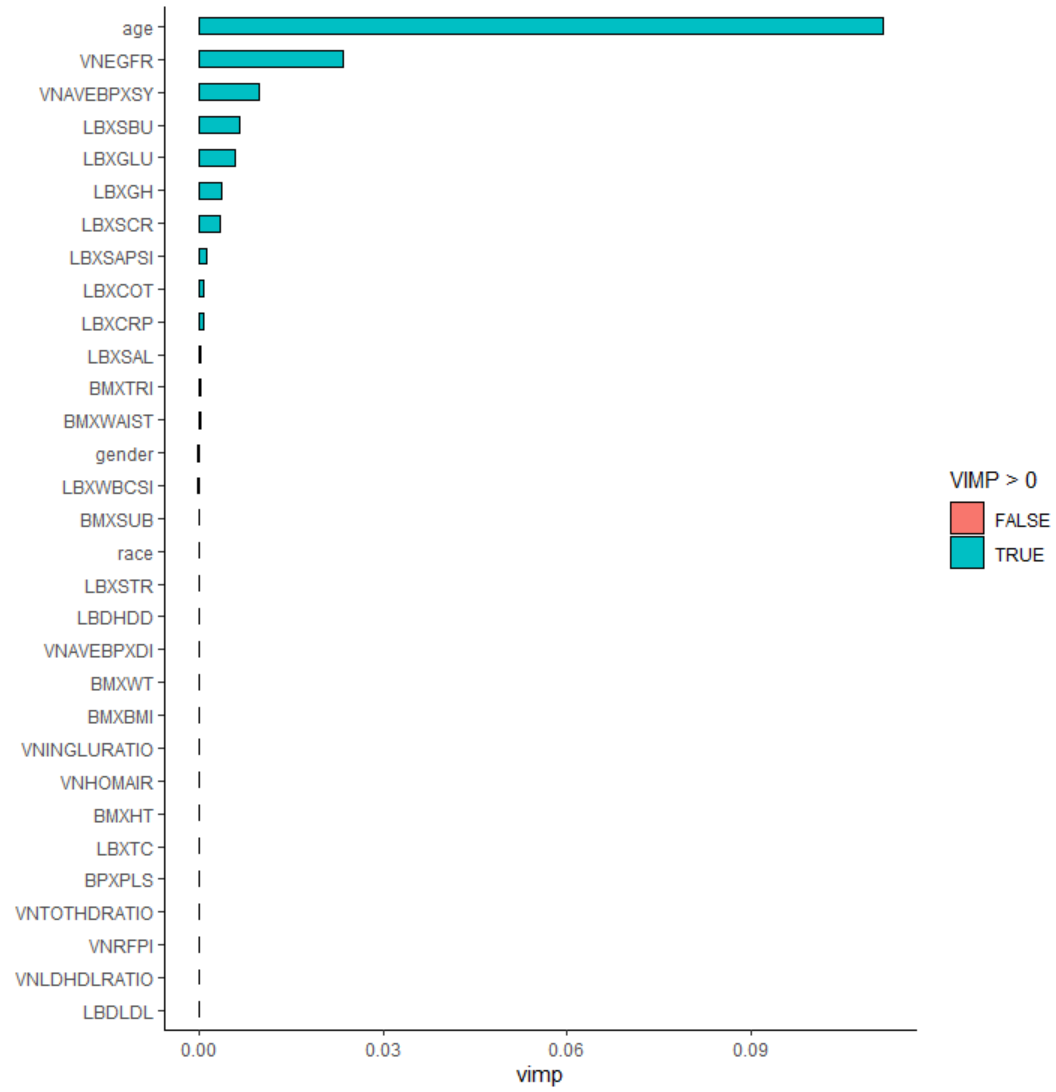


# Fig. S32. The VIMP ranking results for the RSF model using all 28 physiological indicators and 3 demographic indicators with the time to death as the time scale.


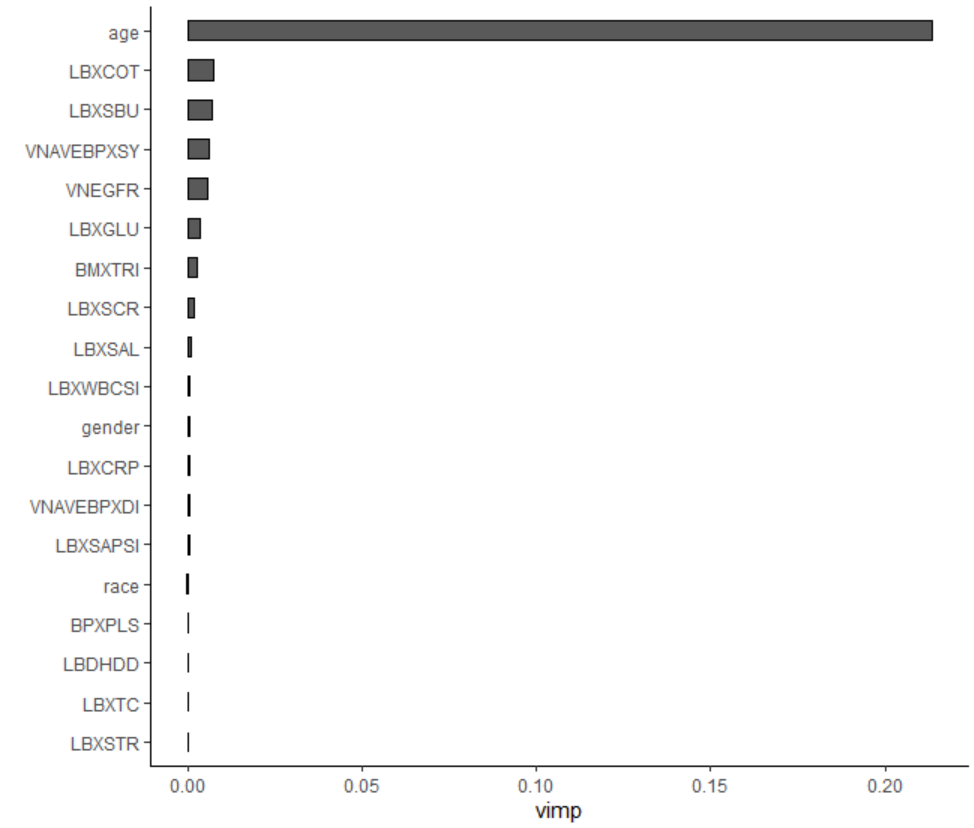


# Fig. S33. The VIMP ranking results for the RSF model using 16 physiological indicators (removing highly correlated variables) and 3 demographic indicators with the time to death as the time scale.

Table S1. Number of participants by mortality status, sex, and race for the entire NHANES population and subpopulations with data for each physiological indicator

| Name | Coding Name | Alive | Deceased | Female | Male | Mexican Americans | Other Hispanics | Non-Hispanic White Americans | Non-Hispanic Black Americans | Other Race/ Multi-Racial |
| --- | --- | --- | --- | --- | --- | --- | --- | --- | --- | --- |
| NHANES Population ^a^ |  |  |  | 41647 (50.73) | 40444 (49.27) | 19161 (23.34) | 6166 (7.51) | 31216 (38.03) | 19400 (23.63) | 6148 (7.49) |
| NHANES Subpopulation ^b^ |  | 40965 (86.67) | 6301 (13.33) | 24486 (51.80) | 22780 (48.20) | 8945 (18.92) | 3453 (7.31) | 21434 (45.35) | 10039 (21.24) | 3395 (7.18) |
| Body Mass Index (kg/m**2) ^c^ | BMXBMI | 38877 (88.26) | 5170 (11.74) | 22770 (51.69) | 21277 (48.31) | 8389 (19.05) | 3232 (7.34) | 19837 (45.04) | 9437 (21.42) | 3152 (7.16) |
| Standing Height (cm) ^c^ | BMXHT | 39043 (88.16) | 5245 (11.84) | 22892 (51.69) | 21396 (48.31) | 8434 (19.04) | 3247 (7.33) | 19949 (45.04) | 9495 (21.44) | 3163 (7.14) |
| Subscapular Skinfold (mm) ^c^ | BMXSUB | 22422 (85.02) | 3952 (14.98) | 13305 (50.45) | 13069 (49.55) | 5681 (21.54) | 1756 (6.66) | 12970 (49.18) | 4850 (18.39) | 1117 (4.24) |
| Triceps Skinfold (mm) ^c^ | BMXTRI | 25357 (85.02) | 4467 (14.98) | 14886 (49.91) | 14938 (50.09) | 6521 (21.86) | 1990 (6.67) | 14255 (47.80) | 5791 (19.42) | 1267 (4.25) |
| Waist Circumference (cm) ^c^ | BMXWAIST | 37695 (88.57) | 4863 (11.43) | 21914 (51.49) | 20644 (48.51) | 8182 (19.23) | 3137 (7.37) | 19198 (45.11) | 9017 (21.19) | 3024 (7.11) |
| Weight (kg) ^c^ | BMXWT | 38933 (88.02) | 5300  (11.98) | 22860 (51.68) | 21373 (48.32) | 8415 (19.02) | 3243 (7.33) | 19954 (45.11) | 9466 (21.40) | 3155 (7.13) |
| Relative Fat Mass Index (-) ^c^ | VNRFPI | 37661 (88.77) | 4764 (11.23) | 21858 (51.52) | 20567 (48.48) | 8165 (19.25) | 3128 (7.37) | 19107 (45.04) | 9004 (21.22) | 3021 (7.12) |
| 60 sec. pulse (30 sec. pulse * 2) ^c^ | BPXPLS | 37837 (87.69) | 5310 (12.31) | 22203 (51.46) | 20944 (48.54) | 8208 (19.02) | 3142 (7.28) | 19605 (45.44) | 9182 (21.28) | 3010 (6.98) |
| Direct HDL-Cholesterol (mg/dL) ^c^ | LBDHDD | 37085 (87.99) | 5060 (12.01) | 21729 (51.56) | 20416 (48.44) | 8103 (19.23) | 3103 (7.36) | 19311 (45.82) | 8653 (20.53) | 2975 (7.06) |
| LDL-cholesterol (mg/dL) ^c^ | LBDLDL | 17411 (88.40) | 2285 (11.60) | 10224 (51.91) | 9472 (48.09) | 3762 (19.10) | 1487 (7.55) | 9048 (45.94) | 3995 (20.28) | 1404 (7.13) |
| Triglycerides (mg/dL) ^c^ | LBXSTR | 36963 (87.97) | 5055 (12.03) | 21651 (51.53) | 20367 (48.47) | 8089 (19.25) | 3092 (7.36) | 19260 (45.84) | 8615 (20.50) | 2962 (7.05) |
| Total cholesterol (mg/dL) ^c^ | LBXTC | 37084 (87.99) | 5063 (12.01) | 21730 (51.56) | 20417 (48.44) | 8103 (19.23) | 3103 (7.36) | 19313 (45.82) | 8653 (20.53) | 2975 (7.06) |
| Diastolic: Average blood pressure (mm Hg) ^c^ | VNAVEBPXDI | 37529 (88.00) | 5118 (12.00) | 21909 (51.37) | 20738 (48.63) | 8127 (19.06) | 3121 (7.32) | 19367 (45.42) | 9037 (21.18) | 2995 (7.02) |
| Systolic: Average blood pressure (mm Hg) ^c^ | VNAVEBPXSY | 37660 (87.82) | 5221 (12.18) | 22037 (51.39) | 20844 (48.61) | 8170 (19.05) | 3133 (7.31) | 19483 (45.44) | 9091 (21.20) | 3004 (7.01) |
| Ratio of LDL to HDL Cholesterol (-) ^c^ | VNLDHDLRATIO | 17411 (88.40) | 2285 (11.60) | 10224 (51.91) | 9472 (48.09) | 3762 (19.10) | 1487 (7.55) | 9048 (45.94) | 3995 (20.28) | 1404 (7.13) |
| Ratio of Total to HDL Cholesterol (-) ^c^ | VNTOTHDRATIO | 37084 (87.99) | 5060 (12.01) | 21728 (51.56) | 20416 (48.44) | 8103 (19.23) | 3103 (7.36) | 19311 (45.82) | 8652 (20.53) | 2975 (7.06) |
| C-reactive protein (mg/dL) ^c^ | LBXCRP | 26719 (84.88) | 4759 (15.12) | 16270 (51.69) | 15208 (48.31) | 6800 (21.60) | 2072 (6.58) | 15051 (47.81) | 6248 (19.85) | 1307 (4.15) |
| White blood cell count (SI) ^c^ | LBXWBCSI | 37510 (87.84) | 5193 (12.16) | 22099 (51.75) | 20604 (48.25) | 8171 (19.13) | 3135 (7.34) | 19524 (45.72) | 8858 (20.74) | 3015 (7.07) |
| Glycohemoglobin (%) ^c^ | LBXGH | 37476 (87.85) | 5181 (12.15) | 22062 (51.72) | 20595 (48.28) | 8171 (19.16) | 3129 (7.34) | 19512 (45.74) | 8825 (20.69) | 3020 (7.08) |
| Glucose, plasma (mg/dL) ^c^ | LBXGLU | 18222 (87.79) | 2535 (12.21) | 10711 (51.60) | 10046 (48.40) | 3987 (19.21) | 1562 (7.53) | 9507 (45.80) | 4241 (20.43) | 1460 (7.03) |
| Alkaline phosphatase (U/L) ^c^ | LBXSAPSI | 32830 (89.15) | 3997 (10.85) | 18967 (51.50) | 17860 (48.50) | 6920 (18.79) | 2874 (7.80) | 16625 (45.14) | 7621 (20.69) | 2787 (7.57) |
| Homeostatic Model Assessment of Insulin Resistance (-) ^c^ | VNHOMAIR | 15241 (86.31) | 2418 (13.69) | 9073 (51.38) | 8586 (48.62) | 3574 (20.24) | 1294 (7.33) | 8179 (46.32) | 3582 (20.28) | 1030 (5.83) |
| Ratio of Insulin to Glucose (uU*dL)/(mg*mL) ^c^ | VNINGLURATIO | 15241 (86.31) | 2418 (13.69) | 9073 (51.38) | 8586 (48.62) | 3574 (20.24) | 1294 (7.33) | 8179 (46.32) | 3582 (20.28) | 1030 (5.83) |
| Albumin (g/dL) ^c^ | LBXSAL | 36983 (87.97) | 5058 (12.03) | 21664 (51.53) | 20377 (48.47) | 8088 (19.24) | 3095 (7.36) | 19271 (45.84) | 8623 (20.51) | 2964 (7.05) |
| Blood urea nitrogen (mg/dL) ^c^ | LBXSBU | 36982 (87.97) | 5056 (12.03) | 21663 (51.53) | 20375 (48.47) | 8088 (19.24) | 3095 (7.36) | 19269 (45.84) | 8623 (20.51) | 2963 (7.05) |
| Creatinine (mg/dL) ^c^ | LBXSCR | 32832 (89.14) | 3999 (10.86) | 18968 (51.50) | 17863 (48.50) | 6920 (18.79) | 2874 (7.80) | 16626 (45.14) | 7623 (20.70) | 2788 (7.57) |
| Cotinine (ng/mL) ^c^ | LBXCOT | 37046 (87.99) | 5056 (12.01) | 21710 (51.56) | 20392 (48.44) | 8090 (19.22) | 3089 (7.34) | 19284 (45.80) | 8652 (20.55) | 2987 (7.09) |
| Estimated Glomerular Filtration Rate (mL/min/1.73 m2) ^c^ | VNEGFR | 32832 (89.14) | 3999 (10.86) | 18968 (51.50) | 17863 (48.50) | 6920 (18.79) | 2874 (7.80) | 16626 (45.14) | 7623 (20.70) | 2788 (7.57) |

^a^ The NHANES population is defined as the sample with data available for age, sex, and race. Mortality data are available for participants who are 18 years or older.

^b^ The NHANES subpopulation is defined as the sample with data available for mortality, age, sex, and race.

^c^ Statistics for each physiological indicator are based on a subpopulation with data available for mortality status, age, sex, race/ethnicity, and the given indicator.

Table S2. Distribution statistics for the NHANES Subpopulation with data for each physiological indicator used in the final model

| Name | Coding Name | Min | 1% quantile | 25% quantile | Median | Mean  (SD) | 75% quantile | 99% quantile | Max |
| --- | --- | --- | --- | --- | --- | --- | --- | --- | --- |
| Age |  | 18 | 18 | 31 | 46 | 47.38  (19.28) | 63 | 85 | 85 |
| White blood cell count (SI) | LBXWBCSI | 1.6 | 3.4 | 5.4 | 6.6 | 6.87  (2.30) | 7.9 | 13.1 | 99.9 |
| Glucose, plasma (mg/dL) | LBXGLU | 36.0 | 72.79 | 91.0 | 98.0 | 106.2  (35.17) | 108.0 | 281 | 587.3 |
| Cotinine (ng/mL) | LBXCOT | 0.011 | 0.011 | 0.019 | 0.060 | 55.63  (123.07) | 12.60 | 509.054 | 1820.00 |
| Estimated Glomerular Filtration Rate (mL/min/1.73 m2) | VNEGFR | 1.854 | 30.808 | 82.017 | 99.587 | 97.899  (25.584) | 116.006 | 151.001 | 193.732 |

**R code used for data analysis**

library("randomForestSRC")

library("ggRandomForests")

library("tidyverse")

library(survival)

library(ggplot2)

library(cowplot)

library(dplyr)

library(MASS)

library(LTRCtrees)

library(rpart.plot)

library(scales)

##############################

#### Load the NHANES DATA ####

##############################

Data <- nhanes_merged_dataset %>%

# Define new columns with more legible codenames

mutate(mortality_status = MORTSTAT

, time_to_death = PERMTH_INT

, weights = WTINT2YR

, cluster = paste(nhanes_merged_dataset$SDMVPSU, nhanes_merged_dataset$SDMVSTRA)

, cycles = SDDSRVYR

, age = RIDAGEYR

, gender = relevel(factor(if_else(RIAGENDR == 1, "_male", "_female"))

, ref = "_male")

, race = relevel(factor(case_when(RIDRETH1 == 1 ~ "_mexican_american"

, RIDRETH1 == 2 ~ "_other_hispanic"

, RIDRETH1 == 3 ~ "_whites"

, RIDRETH1 == 4 ~ "_blacks"

, RIDRETH1 == 5 ~ "_other" ))

, ref = "_whites")) %>%

# Select the pertinent variables after removing highly correlated indicators

dplyr::select("mortality_status", "time_to_death", "age", "gender", "race","BMXBMI","BPXPLS","VNRFPI","VNEGFR","VNHOMAIR","LBXCRP","LBXTC","LBXWBCSI","LBXSAL","LBXSTR","LBXGLU","LBXSAPSI","LBDHDD","LBXSBU","VNAVEBPXSY","VNAVEBPXDI") %>%

# Exclude participants who have missing data

na.omit(.)%>%

# Exclude participants with no follow-up data

filter(time_to_death != 0)

# Create the variable using age as time scale

Data$End <- Data$age + Data$time_to_death/12

Data$Start <- 0

##############################

##### Variable Selection #####

##############################

# Costruct random survival forest model using age as time sclae

set.seed(1)

RSF <- rfsrc(Surv(End, mortality_status) ~ ., data = train,

ntree = 500, mtry=5, nodesize = 2000, importance = T)

# Select needed variables based on VIMP

plot(RSF)

############################################################

##### Visualization of Risk Groups Using Survival Tree #####

############################################################

# Fit survival tree model

set.seed(2)

LTRCART.obj_VNEGFR <- LTRCART(Surv(age,End, mortality_status) ~ gender+ race +VNEGFR,

data = Data, control = rpart::rpart.control(cp = 0.00001, minbucket = 1000))

#Visulaize Survival Tree

rpart.plot(LTRCART.obj_VNEGFR, digits=-2, tweak = 1.2, roundint=FALSE)

############################################################

##### Determination of HR Using Random Survival Forest #####

############################################################

# Construct Random Survival Forest model and calculate the Ensemble Mortality

set.seed(1)

RSF.obj_5 <- rfsrc(Surv(time_to_death, mortality_status) ~ age+ gender+ VNEGFR+ VNRFPI+ LBXGLU+ LBXWBCSI,

data = Data, ntree = 500, mtry=3, nodesize = 200, importance = T)

RSF.obj_5.pred <- predict.rfsrc(RSF.obj_5, Data)

pred.scores <- RSF.obj_5.pred$predicted

pred.scores.norm <- (pred.scores-min(pred.scores))/(max(pred.scores)-min(pred.scores))

cv <- coxph(Surv(time_to_death, mortality_status) ~ pred.scores.norm,

data = cbind(Data[,c(1,2)], pred.scores.norm))

# Calculate the hazard ratio for the reference person

median_person <- Data[1,]

median_person$VNEGFR <- quantile(Data$VNEGFR, .5)

median_person$LBXWBCSI <- quantile(Data$LBXWBCSI, .5)

median_person$LBXGLU <- quantile(Data$LBXGLU, .5)

median_person$VNRFPI <- quantile(Data$VNRFPI, .5)

median_person$age <- quantile(Data$age, .5)

median_person.pred <- predict.rfsrc(RSF.obj_5, median_person)

pred.scores.median_person <- median_person.pred$predicted

median_person.norm <- (pred.scores.median_person - min(pred.scores))/(max(pred.scores)-min(pred.scores))

HR.median_person <- cv$coefficients*median_person.norm

# Design the needed value grids

VNEGFR <- seq(from = quantile(Data$VNEGFR, .05),

to = quantile(Data$VNEGFR, .95),

length.out = 2500)

LBXWBCSI_reference <- rep(quantile(Data$LBXWBCSI, .5), 2500)

LBXGLU_reference <- rep(quantile(Data$LBXGLU, .5), 2500)

VNRFPI_reference <- rep(quantile(Data$VNRFPI, .5), 2500)

gender.list <- unique(Data$gender)

age.list <- c(19, 46, 60, 70, 80)

```

# Calculate the HR on the designed grids

Hazard <- vector()

for (i in 1:5){

for (j in 1:2){

Test <- as.data.frame(cbind(VNEGFR,LBXWBCSI_reference,

LBXGLU_reference,VNRFPI_reference))

Test$age <- rep(age.list[i],2500)

Test$gender <- rep(gender.list[j],2500)

colnames(Test) <- c("VNEGFR", "LBXWBCSI", "LBXGLU", "VNRFPI", "age","gender")

levels(Test$gender) <- levels(Data$gender)

RSF.obj_5.test.pred <- predict.rfsrc(RSF.obj_5, Test)

pred.scores.test <- RSF.obj_5.test.pred$predicted

pred.scores.norm.test <- (pred.scores.test-min(pred.scores))/(max(pred.scores)-min(pred.scores))

HR.test <- cv$coefficients*pred.scores.norm.test

Hazard_norm <- HR.test-HR.median_person

Hazard <- cbind(Hazard,Hazard_norm)

}

}

# Plot the hazard ratio on the test set

Hazard_results <- as.data.frame(cbind(VNEGFR,Hazard))

colnames(Hazard_results) <- c("VNEGFR", "19_male", "19_female", "46_male",

"46_female", "60_male", "60_female", "70_male",

"70_female", "80_male", "80_female")

df <- Hazard_results %>%

gather(key = "variable", value = "value", -VNEGFR)

ggplot(df, aes(x = VNEGFR, y = value)) +

geom_line(aes(color = variable, linetype = variable)) +

scale_color_manual(values = c("darkgray","darkgray", "darkred","darkred",

"blue","blue","darkgreen","darkgreen",

"black","black"))+

scale_linetype_manual(values=c("dashed","solid","dashed","solid", "dashed","solid",

"dashed","solid", "dashed","solid"))+

theme_bw() +

theme(text = element_text(size = 15)) +

theme(axis.text = element_text(size = 15)) +

geom_hline(yintercept = 0, linetype="twodash", color = "red") +

geom_vline(xintercept = quantile(Data$VNEGFR, .5), linetype="twodash", color = "blue") +

xlab("Estimated Glomerular Filtration Rate (mL/min/1.73 m2)") + ylab("log Hazard ratios")

##############################################

##### Visualization of HR Using Heatmaps #####

##############################################

# Set the value grids

VNEGFR <- seq(from = quantile(Data$VNEGFR, .05),

to = quantile(Data$VNEGFR, .95), length.out = 50)

LBXGLU <- seq(from = quantile(Data$LBXGLU, .05),

to = quantile(Data$LBXGLU, .95), length.out = 50)

Test.base <- expand.grid(VNEGFR = VNEGFR, LBXGLU = LBXGLU)

LBXWBCSI.list <- c(quantile(Data$LBXWBCSI, .05),

quantile(Data$LBXWBCSI, .5),

quantile(Data$LBXWBCSI, .95))

VNRFPI.list <- c(quantile(Data$VNRFPI, .05),

quantile(Data$VNRFPI, .5),

quantile(Data$VNRFPI, .95))

# Plot the hazard ratio using heatmaps

plot_list <- list()

count <- 0

for (i in 1:3){

for (j in 1:3){

count <- count+1

age <- rep(age.list[2], 2500)

VNRFPI <- rep(VNRFPI.list[i], 2500)

LBXWBCSI <- rep(LBXWBCSI.list[j], 2500)

Test <- cbind(VNRFPI, Test.base, LBXWBCSI, age)

colnames(Test) <- c("VNRFPI", "VNEGFR", "LBXGLU", "LBXWBCSI", "age")

Test$gender=rep(gender.list[1],2500)

levels(Test$gender) <- levels(Data$gender)

RSF.obj_5.combine.pred.test <- predict.rfsrc(RSF.obj_5, Test)

all.pred.scores.test <- RSF.obj_5.combine.pred.test$predicted

all.pred.scores.norm.test <- (all.pred.scores.test - min(pred.scores))/(max(pred.scores)-min(pred.scores))

HR.all.test <- cv$coefficients*all.pred.scores.norm.test

Hazard_all_norm <- HR.all.test-HR.median_person

p <- ggplot(Test, aes(VNEGFR, LBXGLU, fill=Hazard_all_norm )) +

geom_tile() +

scale_fill_gradient2(low="green", mid="yellow", high="red",

midpoint=0.4,

breaks=seq(-0.4,1.6,0.4),

limits=c(-0.4,1.6))+

theme(legend.position="none")

plot_list[[count]] = ggplotGrob(p)

}

}

cowplot::plot_grid(plotlist = plot_list

**SI References**

1. Gordon, L. and R.A. Olshen, Tree-structured survival analysis. Cancer treatment reports, 1985. 69(10): 1065-1069.
2. Breiman, L., J. Friedman, C.J. Stone, and R.A. Olshen, Classification and regression trees. 1984: CRC press.
3. Ciampi, A., J. Thiffault, J.-P. Nakache, and B. Asselain, Stratification by stepwise regression, correspondence analysis and recursive partition: a comparison of three methods of analysis for survival data with covariates. Computational statistics & data analysis, 1986. 4(3): 185-204.
4. Zhou, Y. and J.J. McArdle, Rationale and applications of survival tree and survival ensemble methods. Psychometrika, 2015. 80(3): 811-833.
5. Ishwaran, H., U.B. Kogalur, E.Z. Gorodeski, A.J. Minn, and M.S. Lauer, High-dimensional variable selection for survival data. Journal of the American Statistical Association, 2010. 105(489): 205-217.
6. Dietrich, S., A. Floegel, M. Troll, T. Kühn, W. Rathmann, A. Peters, D. Sookthai, M. Von Bergen, R. Kaaks, and J. Adamski, Random Survival Forest in practice: a method for modelling complex metabolomics data in time to event analysis. International journal of epidemiology, 2016. 45(5): 1406-1420.
7. Ehrlinger, J. and E.H. Blackstone, ggRandomForests: Survival with Random Forests. 2019.
8. Breiman, L., Random forests. Machine learning, 2001. 45(1): 5-32.
9. Liaw, A. and M. Wiener, Classification and regression by randomForest. R news, 2002. 2(3): 18-22.
10. Ishwaran, H., Variable importance in binary regression trees and forests. Electronic Journal of Statistics, 2007. 1: 519-537.
11. Ishwaran, H., U.B. Kogalur, E.H. Blackstone, and M.S. Lauer, Random survival forests. The annals of applied statistics, 2008. 2(3): 841-860.
